# Surface‐modified protein crowders influence mutant huntingtin exon 1 aggregation via crowding effects, crowder association, and crowder solution stability

**DOI:** 10.1002/pro.70395

**Published:** 2025-11-24

**Authors:** Jakub Haduła, Sabrina T. Krepel, Dhanya Babu, Meng‐Ruo Huang, Arnold J. Boersma

**Affiliations:** ^1^ Cellular Protein Chemistry, Bijvoet Centre for Biomolecular Research, Faculty of Science Utrecht University Utrecht The Netherlands; ^2^ DWI‐Leibniz Institute for Interactive Materials Aachen Germany; ^3^ Structural Biochemistry, Bijvoet Centre for Biomolecular Research, Faculty of Science Utrecht University Utrecht The Netherlands; ^4^ Biomolecular Mass Spectrometry and Proteomics, Bijvoet Centre for Biomolecular Research, Faculty of Science Utrecht University Utrecht The Netherlands

**Keywords:** Förster resonance energy transfer, macromolecular crowding, mutant huntingtin exon 1, protein aggregation

## Abstract

Intracellular protein aggregation occurs in a highly crowded environment. The intracellular environment is highly heterogeneous, featuring diverse crowder protein surface chemistries along with varying crowder stability and solubility. It remains unclear how these aspects influence protein aggregation. Therefore, we assessed how a crowder protein and its surface properties impact aggregation. We utilize high concentrations of surface‐modified proteins based on bovine serum albumin (BSA) to monitor how they influence the aggregation of mutant huntingtin exon 1, enabled by fluorescent proteins (mHttex1‐VC) for förster resonance energy transfer (FRET). This system reveals three mechanisms through which bystander proteins direct mHttex1‐VC aggregation: (1) monodisperse inert proteins appear to function as crowders, increasing the amount of fibrils and their length and width; (2) marginally soluble proteins strongly enhance mHttex1‐VC aggregation and density through coaggregation; and (3) crowders that bind mHttex1‐VC or folding‐destabilized crowders reduce aggregation. The buffer conditions modulate the effects of the protein surface. Thus, in addition to macromolecular crowding effects, the crowder stickiness, solubility, and stability determine the aggregation of the test protein. We expect these effects to also play a role in cells.

## 1 INTRODUCTION

High concentrations of macromolecules found in cells induce macromolecular crowding (Alfano et al., 2024; Monterroso et al., 2024; Rivas & Minton, 2016). The crowders exert steric repulsion, which reduces the available space, also known as the excluded volume. Consequently, it enhances molecular processes that can reduce their volume, such as protein–protein interactions and protein folding. Hence, a well‐described consequence of in‐buffer macromolecular crowding is enhanced protein aggregation (Munishkina et al., 2004; Musiani & Giorgetti, 2017; Patel et al., 2024; Siddiqui & Naeem, 2023; Van Den Berg, 1999). However, the crowding effects on aggregation are challenging to predict due to the complex shapes involved, different reaction pathways, altered solvent properties, and associative interactions with the crowder.

Protein aggregation is associated with neurodegenerative diseases, such as Alzheimer's and Parkinson's disease. Indeed, crowding enhances the formation of the corresponding fibrils (Uversky et al., 2002; Zhou et al., 2009), making it essential to determine the relevance of crowding in intracellular scenarios. Mutations in the gene encoding the huntingtin protein increase the risk of developing Huntington's disease (Tong et al., 2024). Expanding its polyglutamine stretch enhances the formation of truncated huntingtin that consists of the exon 1 domain. More than 35 glutamines (35Q) in the exon 1 domain (mHttex1) leads to protein fibril formation. In patients, fibril formation has a potential causative role in neuronal cell death (Pieri et al., 2012). The toxicity of fibrils depends on their morphology (Jain et al., 2025; Mario Isas et al., 2021), which in turn will be determined by the nucleation structure and pathway, and is affected by many factors such as mHttex1 concentration, point mutations, and chaperones (Boatz et al., 2020; Mishra et al., 2024; Monsellier et al., 2015). Therefore, it is highly relevant to understand how macromolecular crowding affects the aggregation and fibril formation of mHttex1.

Polymeric crowders such as polyethylene glycol (PEG), dextran, and Ficoll are typically used to investigate crowding effects due to their high solubility and relative inertness. However, a macromolecular crowder like PEG exhibits specific behaviors, such as associating with some biomacromolecules (Rivas & Minton, 2022) and partitioning into biomolecular condensates (Alberti et al., 2019; André & Spruijt, 2020; Kanaan et al., 2020). These polymeric crowders have been applied to study the combined effect of crowding and surface interaction on mHttex1 aggregation (Groover et al., 2021), showing that each condition resulted in specific aggregate morphologies as determined by atomic force microscopy. In solution, it was shown that dextran induced liquid‐phase separation of Httex1(Q25), which otherwise did not phase separate (Peskett et al., 2018). These data show that the crowding agent identity determines the outcome of mHttex1 aggregation.

In general, polymers display different concentration regimes as they interpenetrate, which is less relevant to understanding how proteins crowd. The protein content in a cell can vary from 50 to 300 mg/mL (Monterroso et al., 2024; Zimmerman & Trach, 1991), depending on the cell type and the conditions. In addition, 80% of all macromolecules are proteins. Bovine serum albumin (BSA) is the most commonly used protein crowder as it is available in large quantities, is highly soluble, and has a relevant charge density (−18e for a 66 kDa protein). Protein crowding depends on surface chemistry, as weak associative interactions counterbalance steric interactions (Groen et al., 2015; Sarkar et al., 2014). Protein crowding using BSA can dramatically accelerate aggregation, as has, for example, been shown for α‐synuclein (Uversky et al., 2002). In contrast to dextran, BSA did not induce droplets or aggregation of the non‐pathological Httex1 variant with a 25Q stretch (Peskett et al., 2018). Hence, the crowder surface chemistry is important, and BSA is therefore a more relevant crowder than polymers to study the role of crowding proteins.

**FIGURE 1:**
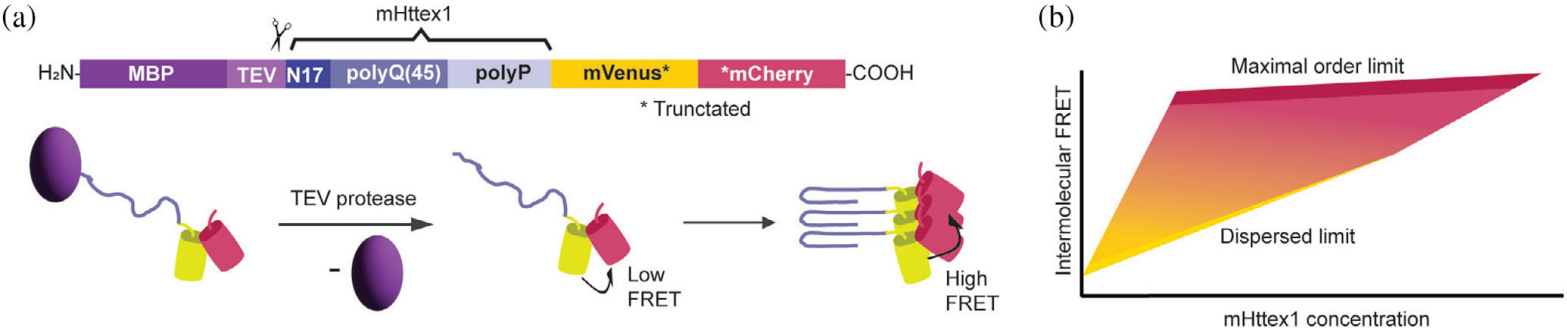
Concept of the mHttex1‐VC sensor to measure aggregate structures. (a) Design of the M‐mHttex1‐VC construct. The linker between mVenus and mCherry is truncated to reduce intramolecular FRET. Aggregation only starts after MBP cleavage with TEV protease. Before aggregation, the FRET from intermolecular FRET is low, but increases upon aggregation. (b) The intermolecular FRET in the aggregate and the solution depends on the measure of order and the concentration of the constructs.

We previously introduced a FRET‐based method to study structural transitions in protein assemblies that allows monitoring the aggregation of mHttex1‐VC in HEK293T and *Saccharomyces cerevisiae* (Wan et al., 2022). This method is based on intermolecular FRET where an mVenus and an mCherry protein are fused to mHttex1‐VC (Figure 1). The native disordered domain between the fluorescent proteins is removed so they cannot move with respect to each other, providing a stable and low intramolecular FRET due to their unfavorable orientation. Hence, we measure intermolecular FRET, which depends on the density and structural order of the aggregate. We observed that the aggregation pathway passed through a low‐order intermediate state before progressing to a fibrillar state, with the lifetime of the intermediate state depending on the host species. This method enables monitoring the structure in single aggregates and assesses the presence of fibrils where foci cannot be resolved. Since most studies that monitor fibril formation rely on ThT dyes, these cannot be applied at relevant concentrations of protein crowder BSA, as ThT binds to it and fluoresces accordingly (Rovnyagina et al., 2018; Sen et al., 2009). Therefore, this intermolecular FRET approach now allows for assessing how protein crowding affects aggregation and fibril formation.

Here, we demonstrate that BSA crowding increases mHttex1‐VC foci formation and the maturation of the foci to a fibrillar state, aligning with macromolecular crowding effects. These crowding effects additionally control fibril morphology. However, chemical modification of the BSA surface and choice of buffer induce two additional relevant mechanisms by which high protein concentrations can affect mHttex1‐VC aggregation: if BSA becomes denatured, it inhibits aggregation likely by directly binding to mHttex1‐VC, while enhancing the BSA agglomeration propensity strongly increases mHttex1‐VC aggregation.

## 2 RESULTS

### 2.1 Macromolecular crowding effect on mHttex1‐VC aggregation

We first fused our previously developed mHttex1‐VC (Wan et al., 2022) with a Tobacco Etch Virus (TEV) protease‐cleavable maltose binding protein (MBP) to be able to control the aggregation reaction (Figure 1). We selected a polyglutamine stretch of 45Q, which is in the pathological range. SDS‐insoluble mHttex1‐VC were not seen by SDS‐PAGE nor foci by fluorescence microscopy directly after the TEV protease reaction, indicating that aggregation does not occur during TEV cleavage. Addition of polymeric crowding agents such as Ficoll PM70, dextran 40 kDa, and PEG 8000 induced diverse aggregation behaviors (Figure S2, Supporting Information), including polymer droplet formation around the aggregates. Therefore, we used BSA as a protein‐based crowder due to its greater relevance to studying how globular proteins induce crowding, as well as its stability, availability, inertness, and solubility.

**FIGURE 2:**
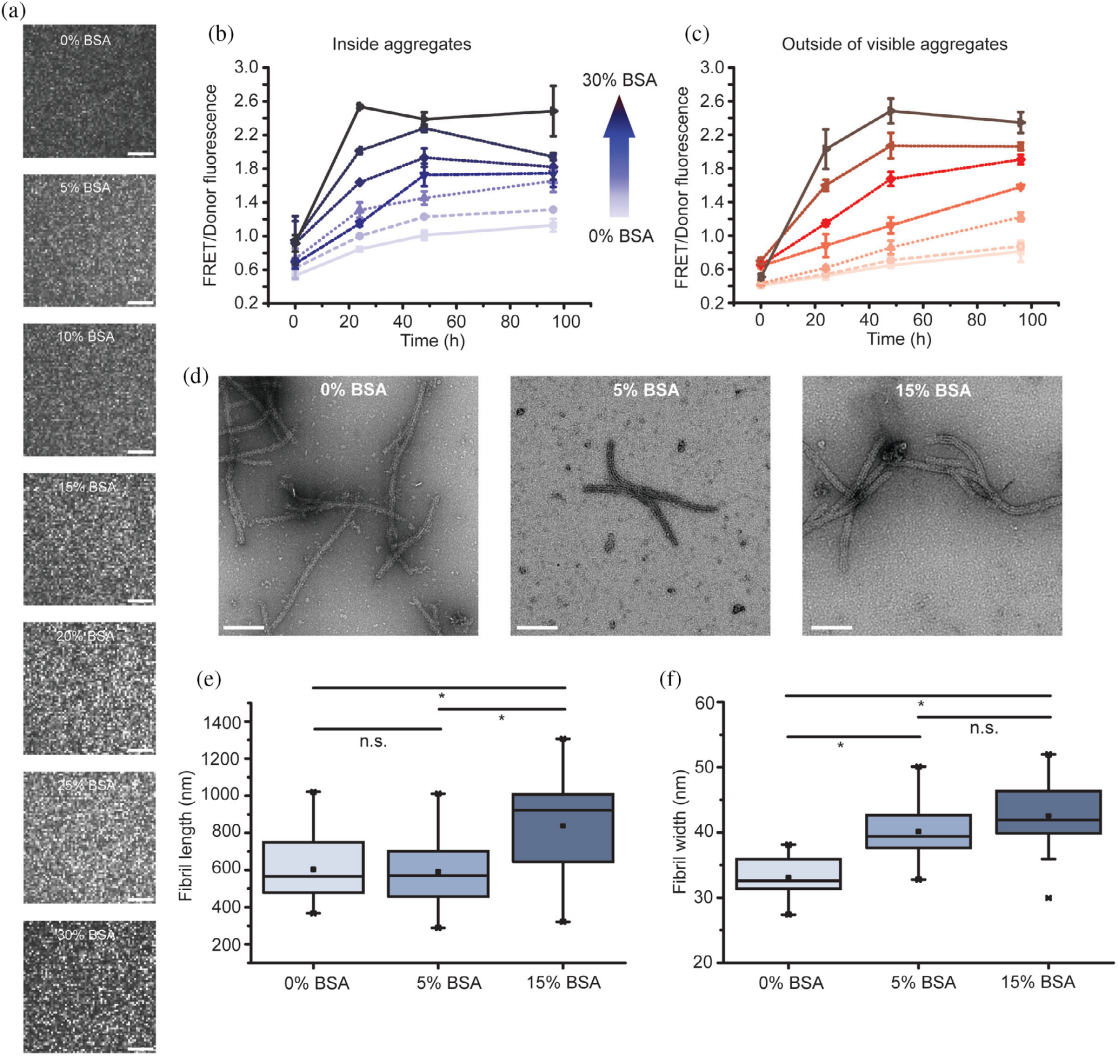
BSA crowding affects mHttex1‐VC aggregate structure progression. (a) Spinning disc microscopy showing increased mHttex1‐VC foci formation with BSA crowding in PBS, pH 7.4 after 96 h. Scale bars = 20 μm. (b) FRET/donor fluorescence progression over time for mHttex1‐VC inside the foci, and the increase in FRET/Donor upon increasing BSA crowding. Average over three biological repeats ±SD, 18 aggregates. (c) As in (b), but outside the visible foci. (d) Negative‐staining electron microscopy images of mHttex1‐VC fibrils formed in different BSA concentrations after 72 h of incubation. Scale bar = 200 nm. (e) mHttex1‐VC fibril length and (f) fibril width as determined from negative‐staining electron microscopy images. Error bars are SD *n* = 30 fibrils. n.s. is *p* > 0.05), * is *p* < 0.00005 (Student's *t* test). BSA, bovine serum albumin.

We first characterized the impact of wild‐type BSA on mHttex1‐VC by spinning disc confocal microscopy. Early work from Uversky et al. showed that 6% w/w BSA increases α‐synuclein aggregation rates (Uversky et al., 2002), whereas Peskett et al. showed the absence of aggregation for mHttex1‐GFP with a 25Q stretch with BSA (Peskett et al., 2018). Here, we observe that mHttex1‐VC with a 45Q stretch does form foci in the presence of BSA crowding (Figure 2a). The mHttex1‐VC concentration in the foci, as inferred from fluorescence intensity, increases over time (Figure S3) as more mHttex1‐VC partitions into the foci. At the same time, mHttex1‐VC content in the dilute phase decreases. To determine the density and order in the aggregates, we measure intermolecular FRET by exciting the donor (mVenus) and measuring the fluorescence from the acceptor (mCherry) and the donor. The ratio of these intensities increases as fibrils or aggregates are formed. Concomitantly, the FRET/donor in the foci increases over time until it reaches a plateau (Figure 2b), similar to what we saw in cells (Wan et al., 2022). The VC without the mHttex1 does not form foci, and its FRET/donor does not change (Figure S3), showing that the mHttex1 domain induces foci formation. The large FRET/donor increase implies that the internal structure of the foci transitions to a, most likely, fibrillar state (Peskett et al., 2018; Wan et al., 2022).

We observed that the eventual FRET/donor ratio correlated with the BSA concentration (Figure 2b). This is not due to an increase in mHttex1‐VC density in the foci, as a relation between fluorescence intensity and FRET/donor could not be resolved (Figure 2c and S3). Instead, the FRET/donor increase must be due to a difference in aggregate structure (see below). The FRET/donor ratios outside the foci increase with a similar dependence on BSA as inside the foci (Figure 2c), albeit they are initially lower and increase more slowly. These are likely fibrils below the resolution of the microscope and may have nucleated in the foci and partitioned into the dilute phase or nucleated outside the foci directly. Hence, BSA crowding increases foci formation, their internal structure, and fibrils outside the foci.

To test our hypothesis that mHttex1‐VC forms fibrillar aggregates and that their structure depends on whether they aggregate in the presence of BSA, as inferred from FRET/donor, we imaged the mixture with negative staining electron microscopy (NS‐EM) (Figure 2d). We see that mHttex1‐VC forms the expected fibrillar bundles with a superstructural heterogeneity common for mHttex1 fibrils (Boatz et al., 2020). Hence, the VC domain does not prevent fibril formation. The fibrils are increasingly clustered with BSA, providing an additional opportunity for FRET. The dimensions of the mHttex1‐VC fibrils also depend on the BSA concentrations: fibril length (Figure 2e) and width (Figure 2f) followed the order 15% BSA > 5% BSA > 0% BSA (*p* = 3.7*10^−5^): fibrils were (8.4 ± 2.3)*10^2^ nm long and 43 ± 3 nm wide when grown in 15% BSA, (5.9 ± 1.7)*10^2^ long and 40 ± 3 nm wide in 5% BSA, and (6.0 ± 1.7)*10^2^ nm long and 33 ± 2 nm wide without BSA. Hence, BSA crowding alters fibril architecture, which is a likely cause for the increased FRET/donor.

**FIGURE 3:**
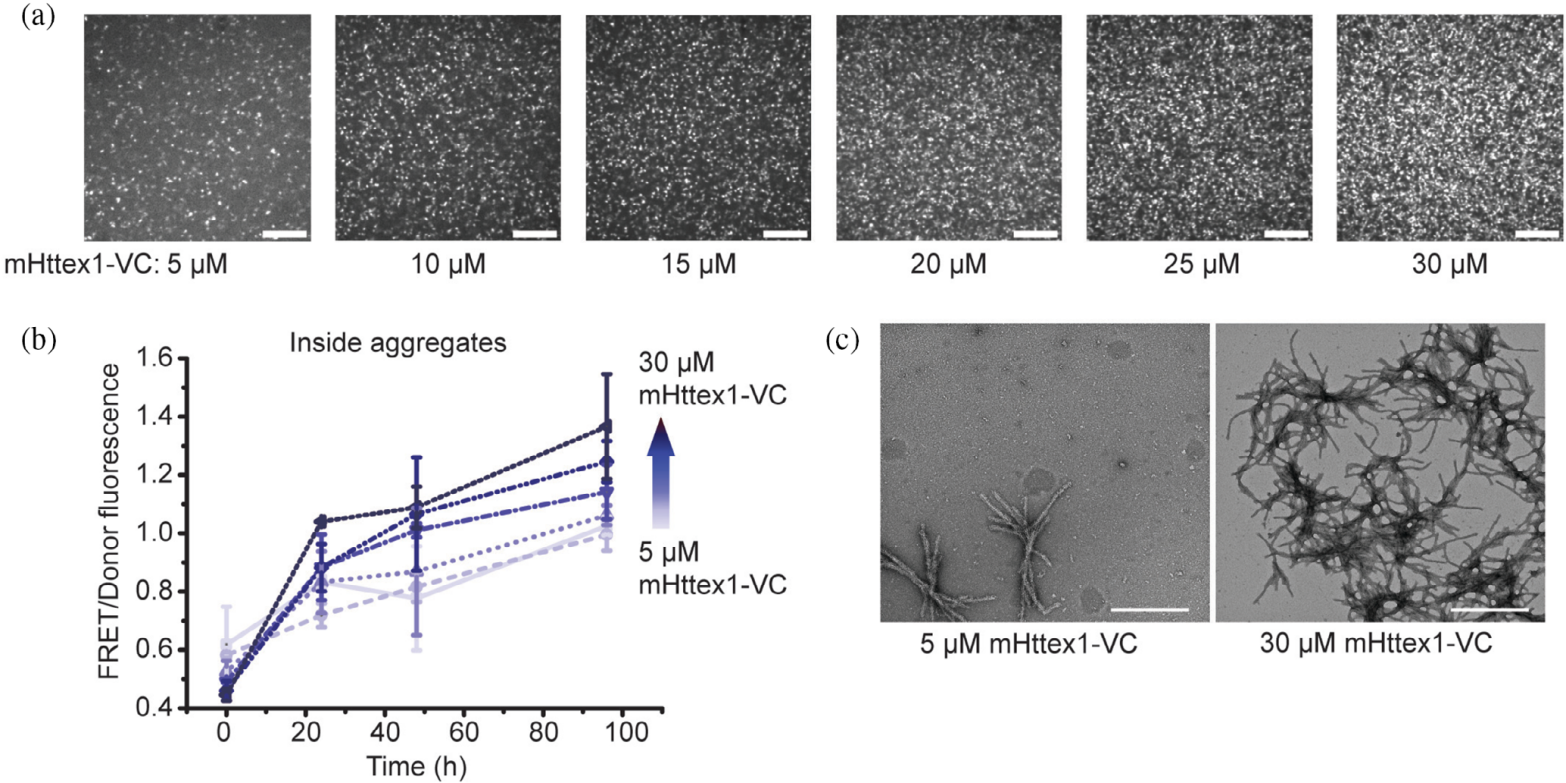
The number of foci and their FRET/donor increases with mHttex1‐VC concentration. (a) Spinning disc confocal microscopy images showing more foci with increasing mHttex1‐VC after 96 h incubation. Scale bar = 20 μm. (b) The increase in FRET/donor of the foci over time for different mHttex1‐VC concentrations. Average over three biological repeats ± SD 18 aggregates. (c) Negative‐staining electron microscopy images of mHttex1‐VC fibrils for two mHttex1‐VC concentrations after 72 h incubation. Scale bar is 500 nm. All measurements are in PBS buffer at room temperature.

Macromolecular crowding increases the thermodynamic activity of the biomacromolecules (Zhou et al., 2008). Therefore, we next determined how much increasing the mHttex1‐VC concentration would replicate the BSA crowding effect. mHttex1‐VC concentration and macromolecular crowders should both increase mHttex1‐VC supersaturation, increasing foci formation. We indeed found that increasing the mHttex1‐VC concentration increased the number of foci (Figure 3a), as it did for BSA. Increasing the mHttex1‐VC concentration increased the FRET/donor of the aggregates, but less so than with BSA crowding (Figure 3b and S4). This means that crowding alters foci maturation differently than increasing the mHttex1‐VC concentration. These observations can be explained with a simplified working model where increasing the concentration of a single‐component system should lead to a larger condensate fraction that retains the same density (Pappu et al., 2023), and we therefore see less change in the foci FRET/donor with mHttex1‐VC concentration. In contrast, repulsive crowder interactions would lead to solvent conditions that compress the condensates to balance the osmotic pressure, thus increasing the FRET/donor in the presence of crowder.

To gain insight into the correlation between FRET/donor and the fibril structure, we imaged the mHttex1‐VC reaction at different concentrations with NS‐EM (Figure 3c). The fibril bundles displayed more branching with mHttex1‐VC concentration than upon increasing the BSA concentration. Although previous measurements have shown that the bundles were wider at higher mHttex1 concentrations (Boatz et al., 2020), we were not able to accurately determine the bundle width as multiple fibril bundles overlapped. The overlap of fibril bundles at higher concentrations gives an additional opportunity for an additional FRET pathway, however.

### 2.2 Protein crowder surface modification alters mHttex1‐VC aggregation

**FIGURE 4:**
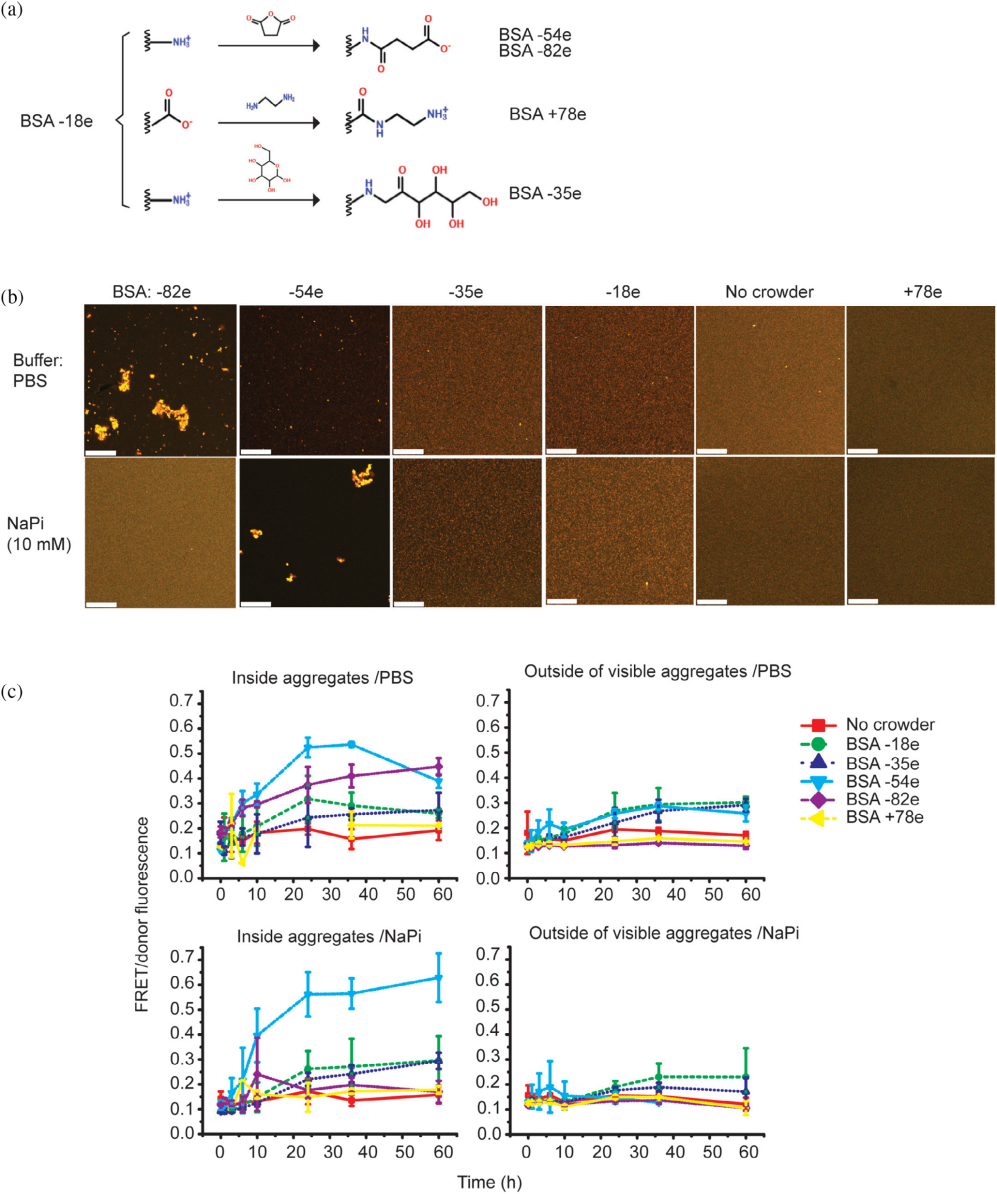
Effect of surface‐charged BSAs on mHttex1‐VC aggregation. (a) Scheme showing chemical modifications of BSA. (b) Scanning confocal microscope images showing mHttex1‐VC aggregates formed after 60 h reaction in different BSAs (15% w/w), dissolved in PBS buffer or 10 mM NaPi, pH 7.4, showing distinct aggregation profiles. Besides the large aggregates of the highly negative BSAs, aggregates were small but abundant and appear here as small bright specks. Scale bar is 36.8 μm. (c) FRET/donor ratios in mHttex1‐VC aggregates and outside of visible aggregates measured over time for different types of BSAs in PBS buffer, pH 7.4 or 10 mM NaPi buffer, pH 7.4. Error bars represent SD, 18 aggregates over three different samples. BSA, bovine serum albumin.

The cytosol hosts diverse crowder surfaces that can interact with an aggregation‐prone protein or with other crowders, thereby counteracting steric crowding effects (Monterroso et al., 2024). To determine the impact of the protein crowder surface, we modified BSA to achieve a large spread of −18e (wild type), −35e, −54e, −82e, and +78e (±3e) net surface charges (Figure 4a), as calculated from the mass from matrix‐assisted laser desorption/ionization‐time‐of‐flight mass spectrometry (MALDI‐TOF) (Figure S5). The samples contain <10% oligomeric species as shown by SDS‐PAGE (Figure S6). Circular dichroism indicates that these BSAs have a similar secondary structure as wild‐type BSA at 0.1 mg/mL (Figure S7). Hence, instead of using other protein crowders such as lysozyme or ovalbumin, we vary the crowder surfaces and retain their size. In addition, we obtain the crowder in large quantities, which is difficult to achieve for protein purified from expression in bacterial cultures.

We followed the aggregation of 10 μM mHttex1‐VC in PBS buffer at pH 7.4 in the presence of the different BSAs by confocal laser scanning microscopy (Figure 4b). The ratiometric values differ from those in the previous sections, as this is a different microscope. BSA −82e and −54e induced bright 1–30 μm‐sized mHttex1‐VC aggregates (Figure S8). At the other extreme, BSA +78e induced sporadic and dim foci. The other BSA variants provided many small foci. The large BSA −82e and BSA −54e aggregates also have a higher FRET/donor ratio (Figure 4c), implying more dense or ordered fibrillar states. The intermediate BSA −18e and BSA −35e aggregates gave intermediate FRET/donor values, while BSA +78e and no crowder gave the lowest FRET/donor values. The FRET/donor inside and outside the visible aggregates were similar in these cases, showing that aggregation occurs below the microscope resolution. In contrast, the aggregates formed in the presence of supercharged BSA −82e and BSA −54e have taken up most of the mHttex1‐VC, depleting the outside phase of fluorescence and lowering the outside FRET/donor. These effects are not due to the VC domain, as we do not see foci for the VC control (Figure S8). Hence, we see a diverse set of mHttex1‐VC aggregation behaviors due to the BSA crowder surface modifications.

To test whether the charge of BSA plays a role, we increased the electrostatic contribution by lowering the ionic strength by using a different buffer. To this end, we dissolved the crowders in 10 mM NaPi buffer instead of phosphate‐buffered saline (PBS). We found that mHttex1‐VC aggregation without crowder was not sensitive to the salt concentration over 3 days, as described in the literature (Peskett et al., 2018). Aggregation in the presence of moderately negative and positively charged BSAs was unaffected by the salt concentration, implying no significant electrostatic contribution to the crowding effect. However, salts strongly influenced the reaction in negatively supercharged BSAs in a complex manner: BSA −54e at lower ionic strength produced large fluorescent agglomerates with a 6‐fold increase in FRET/donor, while the dilute phase was depleted of mHttex1‐VC. Conversely, BSA −82e induced the opposite behavior compared to BSA −54e and reduced aggregation at lower ionic strength (Figure 4c), and is in this respect more similar to +78e. Hence, exchanging PBS with NaPi does not affect moderately charged BSA but alters the aggregation mechanism imposed by the negatively supercharged BSAs.

### 2.3 BSA −82e aggregation drives mHttex1‐VC aggregation

**FIGURE 5:**
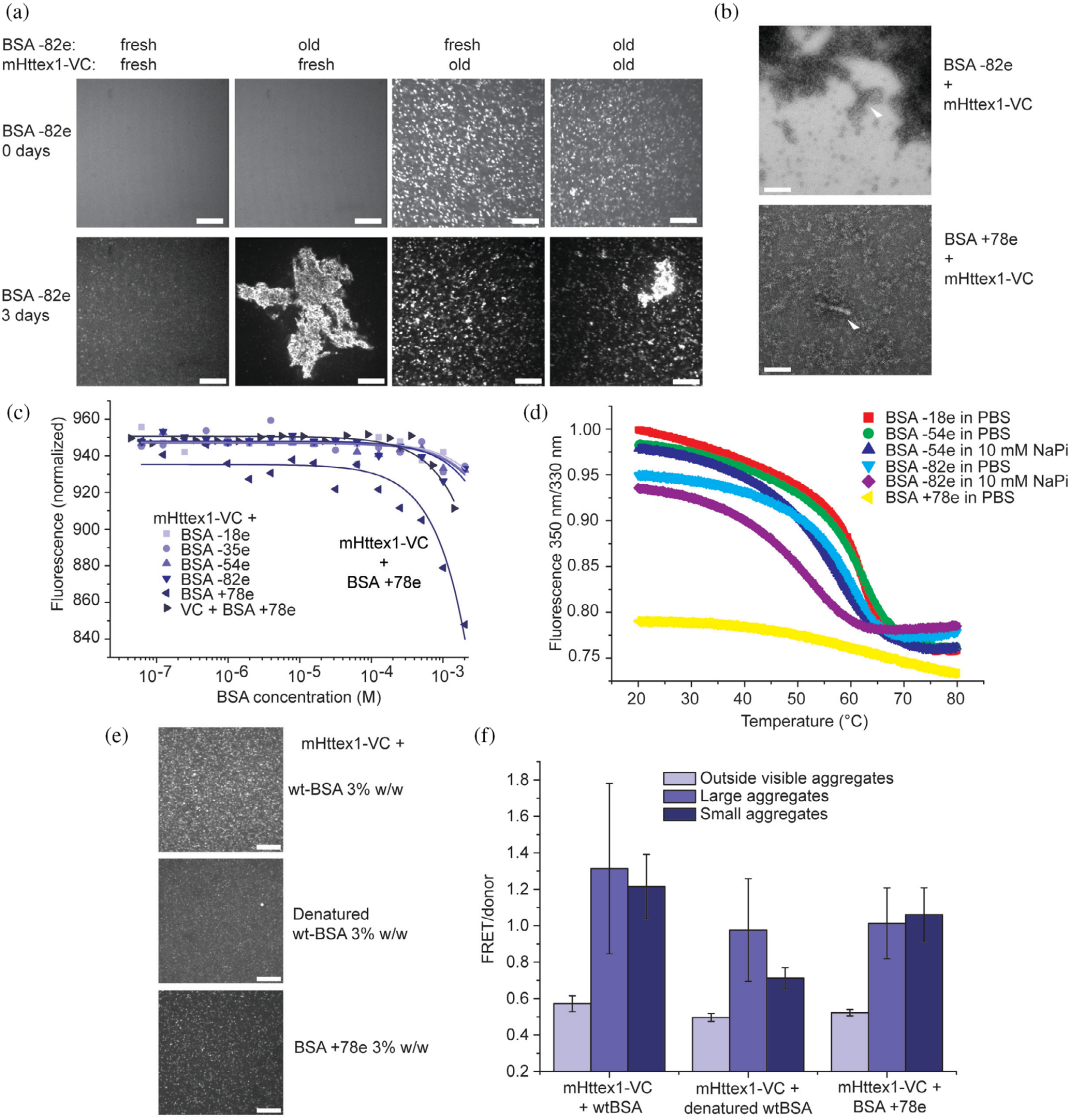
Solution and folding stability of the protein crowder controls mHttex1‐VC aggregation. (a) Spinning disc confocal microscopy images of co‐aggregation of 5% w/w BSA −82e and 10 μM mHttex1‐VC, 3 days after mixing fresh or old solutions. Old solutions were pre‐incubated for 7 days in PBS, pH 7.4. Scale bar is 20 μm. (b) NS‐EM image of mHttex1‐VC in BSA −82e (top) and BSA +78e (bottom) in PBS. White triangles mark representative fibrils. Top: The darker areas are BSA −82e agglomerates/precipitates, which also adhere to the fibril. Scale bar is 200 nm. (c) Microscale thermophoresis to determine binding of the different BSAs to mHttex1‐VC, indicating very weak binding of BSA +78e. (d) 350 nm/330 nm fluorescence versus is in a different state, and BSA −82e has buffer‐dependent compromised stability. Concentration = 2 mg BSA/mL. (e) Spinning disc confocal microscopy images of mHttex1‐VC in wtBSA, heat‐denatured wtBSA, and BSA +78e, all at 3% w/w, imaged after 3 (wtBSA) and 4 (BSA +78e) days. Scale bar is 20 μm. (f) Corresponding FRET/donor emission intensities showing that denatured and +78e BSA (measured after 3 and 4 days) have lower FRET/donor than wtBSA (measured after 3 days). Three biological replicates, 18 aggregates ± SD. Small aggregates are <1.3 μm^2^, large aggregates are >1.3 μm^2^. BSA, bovine serum albumin.

We next investigated why certain conditions could induce the large and high FRET/donor aggregates. We observed by microscopy that BSA −82e in PBS self‐associates over time without adding mHttex1‐VC (Figure S8A). Indeed, DLS analysis generally showed higher polydispersity for conditions where large aggregates are observed (Table S1, Figures S14, S15). These are especially notable for BSA −82e, where species with sizes over the entire detection range of DLS are observed. This is in line with NS‐EM analysis of mHttex1‐VC fibrils formed in the presence of BSA −82e that show that BSA −82e associates with the fibrils and itself, in contrast to wtBSA (Figure 5b). The fibrils are shorter in length, showing that BSA −82e affects the aggregation process. These observations led us to hypothesize that the self‐association propensity of BSA −82e enhances mHttex1‐VC aggregation, as aggregated BSA −82e could provide a surface for heterogeneous nucleation. To test this hypothesis, we used aggregate‐containing solutions of BSA −82e and mHttex1‐VC that were preincubated separately for 7 days. The aggregate‐containing solutions were mixed with freshly prepared solutions to obtain 10 μM mHttex1‐VC in 5% BSA −82e with fresh:fresh, fresh:old, old:fresh, and old:old mHttex1‐VC:BSA −82e. These samples were analyzed after 4 days using spinning disc microscopy (Figure 5a). In contrast to mixing fresh samples or applying old wtBSA (Figure S9), we observed the large aggregates when fresh mHttex1‐VC was incubated with old aggregated BSA −82e. This means that BSA −82e aggregates indeed assist in mHttex1‐VC aggregation. Combining old and aggregated mHttex1‐VC with BSA −82e induced the assembly of larger aggregate structures. This did not happen for wtBSA (Figure S9), showing that BSA −82e also impacts the clustering of preformed fibrils. The FRET/donor ratio in the aggregates equilibrated after 74 h, despite the age difference between the aggregates (Figure S10). Hence, the marginal solution stability of BSA −82e drives large mHttex1‐VC aggregate formation through co‐aggregation.

### 2.4 Crowders that inhibit mHttex1‐VC aggregation are sticky and bind mHttex1‐VC

To explain the inhibitory nature of some crowding conditions, we first analyzed BSA +78e. We hypothesized that BSA +78e prevents aggregation by binding to mHttex1‐VC. We measured the binding affinities with microscale thermophoresis using the VC domain as a fluorescent label (Figure 5b). To prevent aggregation, the measurement was performed within 1 h after cleaving the maltose‐binding protein from mHttex1‐VC. Upon titrating the different BSAs, we indeed detected weak apparent binding of BSA +78e to mHttex1‐VC. The affinity is too low for quantification due to a lack of binding saturation, and the Kd will be >1 mM. As a control for viscosity and fluorescence artifacts, we tested the VC control (Figure S11). We observed a minor decrease, indicating that artifacts do not play the dominant role. We infer that BSA +78e binds to mHttex1, and this is a likely mechanism to reduce aggregation.

NS‐EM analysis showed some BSA +78e associated with the sporadic mHttex1‐VC fibrils (Figure 5b), consistent with the enhanced affinity. In addition, no significant agglomeration was detected as in the case of the BSA −82e aggregates. While heat‐denatured BSA has also been shown to form fibrils (Holm et al., 2007), the fibrils here consisted of mHttex1‐VC, as fibrils were not found in its absence. To further characterize the BSA +78e, we measured the 350/330 nm fluorescence ratio to assess thermal unfolding (Figure 5c). BSA +78e displayed a lower ratio and the lack of an apparent thermal unfolding step, which was present in all other BSAs. Hence, BSA +78e associates with mHttex1‐VC and is in a different state than the other BSAs.

We investigated the dimensions of the BSAs with dynamic light scattering (DLS), showing that BSA +78e had a much larger radius than the others (16 vs. 4–5 nm) (Table S1). Since 16 nm is too large for unfolded BSA +78e, we infer that it forms oligomers. Oligomerization is in line with the lower 350/330 nm fluorescence ratio that is typically associated with tryptophans occupying a more hydrophobic environment. Circular dichroism (CD) spectrometry shows that BSA +78e remains folded, at least when highly diluted, albeit its folding may be concentration‐dependent as in the case of denatured BSA (see below). Thus, in addition to enhanced interaction with mHttex1‐VC, BSA +78e also exhibits higher self‐interaction.

We hypothesized that heat‐denatured BSA would also exhibit increased stickiness and, therefore, be able to reduce mHttex1‐VC aggregation. Denatured oligomeric wtBSA has been proposed to be more sticky than natively folded BSA (Park et al., 2018; Shirahama & Suzawa, 1988). Heat‐denatured BSA forms irreversible β‐structure‐containing oligomers stabilized by disulfide bonds (Kalacheva et al., 2025; Wetzel et al., 1980). CD indicated a concentration‐dependent denaturation of heated BSA after cooling (Figure S7). We performed the experiments in 3% w/w BSA and 10 mM NaPi buffer, which provided monodisperse oligomeric species of denatured wtBSA and prevented gelation (Kalacheva et al., 2025). Indeed, heat‐denatured BSA oligomers reduced aggregation of mHttex1‐VC, resulting in smaller and fewer aggregates with a lower FRET/donor than in the presence of folded BSA (Figure 5e,f). For equal comparison, BSA +78e at 3% w/w also reduced aggregation. To test for the dependence of FRET on aggregate size, we categorized the aggregates. We observed no consistent relationship between aggregate size and FRET.

As BSA −82e in NaPi buffer also inhibited aggregation, we hypothesized a similar mechanism. BSA −82e in NaPi had a higher tendency to thermally unfold and aggregate compared to PBS buffer (Figure 5d), possibly due to enhanced intramolecular electrostatic repulsion. These measurements are conducted under dilute conditions, and the BSA −82e stability is likely to be further reduced when highly concentrated (Wetzel et al., 1980).

## 3 DISCUSSION

While the effect of macromolecular crowding on protein aggregation has been extensively studied, the focus has mainly been on polymer‐based crowders, likely due to their compatibility with, for example, ThT dyes used for fibril detection. Only occasional attention has been paid to protein‐based crowders. Moreover, how the surface chemistry, the folding state, and the solubility of these crowders affect aggregation has been unclear. In this study, we employ FRET to differentiate the structural properties of the aggregates, demonstrating that BSA influences mHttex1‐VC differently than conventional polymeric crowders. The high protein concentration induces three distinct pathways depending on the surface chemistry of the protein crowders: inert crowders enhance mHttex1‐VC aggregation and increase aggregate density, including fibril length and width, through macromolecular crowding effects; marginally soluble crowders coaggregate with mHttex1‐VC, thereby increasing aggregation and aggregate density; and crowders that associate with mHttex1‐VC reduce aggregation.

The fluorescent proteins will influence the results described here as they are known to change the eventual aggregate structures (Riguet et al., 2021). However, the identified principal mechanisms by which high concentrations of bystander protein can affect the aggregation of a test protein should remain valid. We do not expect the cleaved MBP and the TEV protease to impact the reaction, as the BSA is in 500× excess and only 10 μM MBP is generated. While polymeric crowders are absent in cells and their effects are less relevant, BSA is also not found in cells. Nonetheless, its shape and surface chemistry are more appropriate for studying globular protein crowding in cells than those of polymers.

Here, all aggregation pathways started with low FRET/donor foci that are, therefore, less structured or amorphous. Previous work identified stepwise mHttex1 aggregation pathways from the well‐established mechanism of starting from alpha‐helix forming mHttex1 oligomers, followed by fibril formation (Jayaraman et al., 2012; Mishra et al., 2024), or, more recently, from a biomolecular condensate (amorphous phase) to a fibril phase (Peskett et al., 2018; Posey et al., 2018; Wan et al., 2022). Fluorescent proteins have been shown to promote phase separation and may thereby enhance the phase separation mechanism (Pandey et al., 2024). However, these mechanisms are not mutually exclusive, as alpha‐helix‐induced oligomer formation is concentration‐dependent (Mishra et al., 2024), and should thereby be accelerated in the concentrated condensate. The macromolecular crowding may further increase the density in the visible assemblies through osmotic compression, thereby accelerating the fibrillation reaction locally.

In cells, we previously observed with the same FRET system that foci in *S. cerevisiae* and HEK293T progressed from a low‐FRET amorphous phase to a high‐FRET fibril state (Wan et al., 2022). In cells, we see that the dwell time of the amorphous phase was species‐dependent. Here, we consistently see a similar profile that starts with a low FRET state, further emphasizing that the reaction occurs as a two‐step process. With the conditions described here, we span a wide range of FRET/donor changes that can exceed the 2–3× increase in FRET/Donor for *S. cerevisiae* and 3–4× increase for HEK293T. The modified proteins can thus replicate in vivo results based on FRET. This may however be a coincidence, as many other factors should play a significant role as well.

Crowder solubility and crowder folding stability are effects that remain hidden when using polymeric crowders and, to the best of our knowledge, have not been assessed in the context of macromolecular crowding effects on test proteins. These effects follow BSA stability as judged by their melting temperatures (Figure 5d). While the BSA radius and hence the excluded volume align with our observations to some extent, it does not account for the buffer effects. Crowding can increase all steps along the pathway (Horvath et al., 2021), and we indeed observe an increased number of amorphous assemblies, along with a faster increase in fibrillation. Modulations in macromolecular crowding effects are typically explained by weak associative or repulsive interactions that balance purely steric macromolecular crowding effects. Indeed, crowders can increase or decrease aggregation (Gorensek‐Benitez et al., 2022). In this view, associative interactions would reduce aggregation in the presence of BSA +78e, denatured BSA, and BSA −82e in NaPi buffer, which we also find here. Indeed, it has been previously shown that BSA can inhibit the fibrillation of other proteins (Finn et al., 2012), reiterating the well‐known phenomena that crowder inertness is test protein‐dependent. Repulsive interactions would increase aggregation for BSA −82e in PBS buffer and −54e; however, enhanced aggregation when employing old BSA −82e presents co‐aggregation as an alternative pathway.

BSA −82e/PBS and −54e/NaPi co‐aggregate with mHttex1‐VC. mHttex1 has been shown to co‐aggregate with various partners in cells (Ormsby et al., 2020; Riguet et al., 2021), and with TDP‐43 (George et al., 2024). In the latter case, mHttex1 aggregation is reduced in the presence of disordered TDP‐43. Our mechanism is likely different, as we observe enhanced aggregation. Based on the melting temperatures and the CD, we assume that a significant portion of these BSAs remains folded during a process called agglomeration (Garcia‐Seisdedos et al., 2019). Self‐assembly of folded proteins can be aided by hydrophobic patches: BSA is relatively hydrophobic for a globular protein (Cardamone & Puri, 1992), and its hydrophobic patches may be more exposed due to surface modification‐induced local destabilization. In addition, cations in solution could bridge the negative charges, further promoting self‐assembly. Possibly, the association of mHttex1‐VC with the surfaces presented by marginally stable BSA stabilizes its N‐terminal alpha‐helical conformation, or stabilizes the critical nucleus size, similar to the increased propensity of the amphipathic N‐terminal peptide to bind lipid membranes (Burke et al., 2013).

Reducing BSA stability through heat denaturation or cationizing its surface results in oligomers with a hydrodynamic radius of ~16 nm. The binding of cationized BSA with mHttex1‐VC suggests that denatured BSA may also reduce aggregation through binding. Binding its target would inhibit aggregation, similar to the action of a holdase chaperone. The crowders may bind different assembly species along the aggregation pathway, thus preventing nucleation or elongation and effectively counteracting the crowding. Another explanation may involve increased viscosity, which is known to reduce protein aggregation (Uversky et al., 2002). However, the inhibition of aggregation occurs at only 3% w/w unfolded crowder, while 30% w/w folded crowder still enhances protein aggregation despite the increase in viscosity. Therefore, association with mHttex1‐VC appears to be a more plausible mechanism, although we cannot rule out a contribution from viscosity.

These effects should be relevant in cells since many proteins are marginally stable (Ghosh & Dill, 2010; Van Gils et al., 2022), and a significant part of the proteome loses solubility during aging (David et al., 2010). Especially under conditions where the capacity for protein homeostasis declines, such as during aging (Hipp et al., 2019), faulty bystander proteins could either accelerate aggregation through coaggregation or inhibit aggregation through binding. BSA forms a specific species upon denaturation, and it is unclear if other proteins form similar species. Nonetheless, the eventual outcome would reflect a balance between these opposing effects in a background of macromolecular crowding effects.

## 4 MATERIALS AND METHODS

### 4.1 Materials

All chemicals were purchased from Sigma‐Aldrich, unless mentioned otherwise.

### 4.2 M‐mHttex1‐VC and VC cloning and expression

The MBP‐mHttex1‐mVenus/mCherry (M‐mHttex1‐VC) (45Q) and mVenus/mCherry (VC) genes (GeneArt) were subcloned into pRSET A. *Escherichia coli* BL21(DE3) was transformed with the plasmids, and single colonies were picked to inoculate Luria‐Bertani (LB) broth, which was incubated at 37°C overnight. The precultures were diluted in 1 L Terrific broth (TB) to provide OD_600_ = 0.1 and shaken at 200 rpm at 37°C until reaching OD_600_ = 0.6. The culture was cooled to 18°C, and expression was induced with 0.1 mM isopropyl‐β‐D‐thiogalactoside (IPTG). The culture was shaken overnight at 18°C, after which the cells were spun down, and the supernatant was removed.

### 4.3 M‐mHttex1‐VC and VC purification

The bacterial pellets containing MBP‐mHttex1‐VC were resuspended in 20 mM Tris–HCl, pH 7.4, with 300 mM NaCl and lysed by EmulsiFlex‐C5 cell disruptor (Avestin). The lysates were loaded on a Ni‐NTA column, washed with 50 mM Tris–HCl, 300 mM NaCl, 20 mM imidazole, pH 7.4, and eluted with the same buffer with 400 mM imidazole added. The fractions containing the most protein were collected and loaded on a Superdex 200 10/300 GL Size Exclusion Chromatography (Cytiva) column attached to the Äkta Pure system (Cytiva). The proteins were further purified in PBS, pH 7.4. During purification, protein levels were monitored at wavelengths of 280 nm, 515 nm (mVenus), and 587 nm (mCherry). The purity of collected fractions was analyzed on 12% SDS‐PAGE (Figure S1). Pure fractions were stored at −80°C.

### 4.4 Synthesis of surface‐modified BSAs

BSAs were modified following published procedures: negative BSAs (−54e and −82e) were obtained by reaction with succinic anhydride (Chu et al., 1982), negative BSA (−35e) by glucosylation (Lapolla et al., 1993) and positive BSA (+78e) by reaction with ethylenediamine (Tayyab & Qasim, 1986). BSA was modified with different molar ratios of reagents to BSA, resulting in changes to the protein's surface charge. With a succinic anhydride/BSA ratio of 80, 18 amino groups on BSA were modified, giving a most common surface charge of −54e. With a ratio of 250, 32 amino groups were modified, giving the most common surface charge of −82e. The molecular mass of the main species for each BSA was determined by MALDI‐TOF.

### 4.5 mHttex1‐VC aggregation assays

TEV protease was added to cleave the MBP from mHttex1‐VC, initiating aggregation, to 40 μM mHttex1‐VC in a ratio of 1/20 (w/w). The mixture was left for 30 min at room temperature for complete MBP cleavage, as observed by SDS‐PAGE. Next, the mixture was added to 1.5 mL Eppendorf tubes containing a solution of the corresponding macromolecular crowders to achieve the desired mHttex1‐VC and crowder concentrations, reaching a final volume of 100 μL.

### 4.6 Confocal laser scanning microscopy

The sample (10 μL) was applied on a glass slide, covered with a cover slide, and visualized with a Leica SP8 confocal scanning microscope (63× water immersion objective) and imaged at room temperature. The donor (mVenus) was excited at 488 nm, and the acceptor (mCherry) was excited at 561 nm. The emission was recorded between 505–555 nm (mVenus) and 600–700 nm (mCherry). The sample was imaged in the middle, between the glass surfaces. The images of the donor (excitation and emission of the donor), FRET (excitation of the donor and emission of the acceptor), and acceptor (excitation and emission of the acceptor) were collected and analyzed using ImageJ software. The background of the corresponding solutions with fluorescent proteins was subtracted before analysis. Foci and fluorescence outside were selected manually, and their ratios were determined with the same selection in each fluorescence channel. Fluorescence outside foci was carefully selected to prevent incorporation of out‐of‐focus foci. Foci ratios were highly homogeneous within a sample, and therefore, analysis of six foci per time point was sufficient. This was repeated for three biological repeats.

### 4.7 Spinning disc confocal microscopy

We used a spinning disc confocal microscope to capture faster diffusing foci. The custom‐built microscope was an Eclipse Ti2‐E with PFS (Nikon, Japan), stage MS‐2000‐XYZ with Piezo Top Plate (ASI, USA), a spinning disc unit CSU‐W1‐T1 (Yokogawa, Japan), objective Apo λD 60×/1.42, WD 0.15, oil, MRD71670 (Nikon, Japan), 8.83 pix/μm or 113 nm/pix, lasers Vortran Stradus 488 (150 mW) (Vortran, USA) and Coherent OBIS 561 nm (150 mW) (Coherent, USA), beam splitter OptoSplit II two‐channel for simultaneous colocalization (Cairn Research, UK) with filters ET525/50 m (GFP), ET630/75 m (mCherry) and T585lpxr dichroic (Chroma, USA). The sample was measured and analyzed as described above.

### 4.8 Protein stability assays

The protein stability assay consisted of the F_350_/F_330_ fluorescence ratio, turbidity, and DLS (NanoTemper© Prometheus Panta). BSAs (2 mg/mL) in PBS, or 10 mM NaPi, pH 7.4, were aspirated into High‐Sensitivity Prometheus Panta capillaries. The sample was subjected to a temperature window from 20°C to 70°C to measure protein stability.

### 4.9 Microscale thermophoresis

Micro‐scale thermophoresis (NanoTemper© Monolith) was used to determine BSA binding to mHttex1‐VC. About 10 μM mHttex1‐VC (target) with different concentrations of BSA (ligand) was aspirated into capillaries (Monolith). The binding was measured based on changes in the fluorescence of mCherry in mHttex1‐VC upon potential BSA binding.

### 4.10 Circular dichroism measurements

CD spectroscopy measurements were carried out on a JASCO J‐810 spectropolarimeter with a 1 mm path length quartz cuvette. CD spectra of native BSA (−18e), BSA (−35e), BSA (−54e), BSA (−82e), and BSA (+78e) and denatured native BSA were recorded at 0.1 and 0.5 mg/mL concentrations. Denatured BSA was prepared by heating native BSA (6–7 mg/mL) at 95°C for 5 min and diluted to either 0.1 or 0.5 mg/mL. Every measurement consisted of 10 accumulations, and the following measurement parameters were used: absorption bandwidth 1 nm; scanning speed 100 nm/min; data pitch 0.1 nm.

### 4.11 Negative staining electron microscopy

NS‐EM samples were prepared by pipetting 3 μL of the respective sample onto a glow‐discharged (30 s, 10 mA), 400 mesh carbon‐coated copper grid, incubated for 60 s at room temperature, and manually blotted away. After washing twice with MiliQ water, grids were subsequently stained with 2% (w/v) uranyl acetate (Biolyst Scientific Electron Microscopy Sciences) by submerging the top of the grid and immediately blotting away excess stain twice and once after incubating for 60 s. Samples were imaged on a Talos L120C electron microscope (Thermo Fisher Scientific) operating with a 4 k × 4 k Ceta CMOS camera (Thermo Fisher Scientific). Fibril length and width were analyzed using ImageJ software.

## AUTHOR CONTRIBUTIONS

**Jakub Haduła:** Investigation; writing – original draft; methodology; validation; visualization; writing – review and editing; formal analysis. **Sabrina T. Krepel:** Investigation; methodology; writing – review and editing; formal analysis. **Dhanya Babu:** Investigation; methodology; formal analysis; writing – review and editing. **Meng‐Ruo Huang:** Resources; writing – review and editing. **Arnold J. Boersma:** Conceptualization; writing – review and editing; project administration; supervision; visualization; funding acquisition.

## Supporting information


**Data S1:** Supplementary information.

## Data Availability

The data that support the findings of this study are available from the corresponding author upon reasonable request.
