# Supplementary material for: Surface‐modified protein crowders influence mutant huntingtin exon 1 aggregation via crowding effects, crowder association, and crowder solution stability
Source: Protein Sci. 2025 Nov 24;34(12):e70395. doi: 10.1002/pro.70395 (PMC12641570; doi:10.1002/pro.70395)
Supplement: Supplementary file 1 — Data S1: Supplementary information. [file PRO-34-e70395-s001.docx]

**Supporting Information**

**Surface-modified protein crowders influence** **mutant huntingtin exon 1 aggregation via crowding effects, crowder association, and crowder solution stability.**

Jakub Hadula ^[1][4]^, Sabrina T. Krepel^[2][3]^, Dhanya Babu^[1]^, Meng-Ruo Huang^[4]^, Arnold J. Boersma*^[1][4]^

^[1]^ Cellular Protein Chemistry, Bijvoet Centre for Biomolecular Research, Faculty of Science, Utrecht University, Utrecht 3584 CH, The Netherlands
^[2]^ Structural Biochemistry, Bijvoet Centre for Biomolecular Research, Faculty of Science, Utrecht University, Utrecht 3584 CG, The Netherlands
^[3]^ Biomolecular Mass Spectrometry and Proteomics, Bijvoet Centre for Biomolecular Research, Faculty of Science, Utrecht University, Utrecht 3584 CH, The Netherlands

^[4]^ DWI-Leibniz Institute for Interactive Materials, Aachen 52074, Germany

|  | Cumulant Radius (nm) | | Cumulant PDI | |
| --- | --- | --- | --- | --- |
| Buffer and BSA | Average ⌀ | St. deviation σ | Average ⌀ | St. deviation σ |
| PBS +78e | 16.41 | 0.37 | 0.37 | 0.04 |
| PBS -18e | 4.29 | 0.21 | 0.10 | 0.05 |
| PBS -35e | 4.42 | 0.06 | 0.05 | 0.02 |
| PBS -54e | 4.89 | 0.05 | 0.14 | 0.00 |
| PBS -82e | 5.36 | 0.13 | 0.42 | 0.04 |
| NaPi +78e | 16.13 | 0.06 | 0.34 | 0.01 |
| NaPi -18e | 4.19 | 0.06 | 0.05 | 0.01 |
| NaPi -35e | 4.29 | 0.02 | 0.04 | 0.01 |
| NaPi -54e | 5.03 | 0.04 | 0.20 | 0.02 |
| NaPi -82e | 5.59 | 0.07 | 0.44 | 0.03 |

**Table S1. Dynamic light scattering measurements of different BSAs in PBS or 10 mM NaPi, pH 7.4**. BSAs (2 mg/mL) were loaded in NanoTemper© Prometheus Panta capillaries, and DLS was measured at 20 °C. Modification of BSA changes its cumulant hydrodynamic radius and cumulant polydispersity Index (PDI).


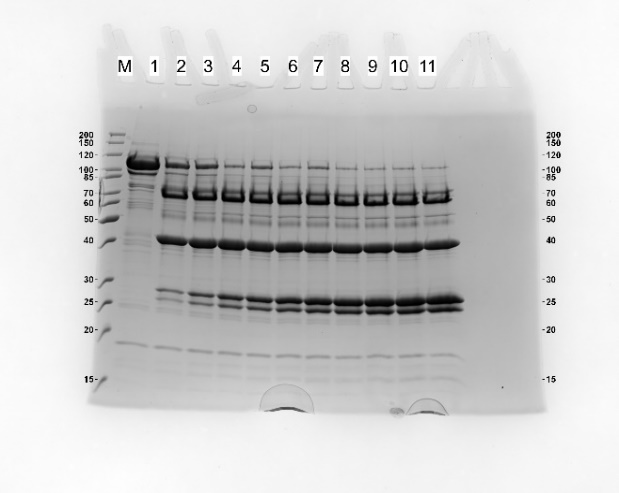


**Fig. S1.** **12% SDS-PAGE gel of MBP-mHttex1-VC treated by TEV protease.** Measured after 30 minutes in different ratios MBP-mHttex1-VC: M – marker, 1 – no TEV protease, 2- 20:1, 3 – 20:2, 4 – 20:3, 5 – 20:4, 6 – 20:5, 7 – 20:6, 8 – 20:7, 9 – 20:8, 10 – 20:9, 11 – 20:10 . MBP-mHttex1-VC: 106 kDa, MBP: 42 kDa, mHttex1-VC: 64 kDa, TEV protease: 27 kDa.


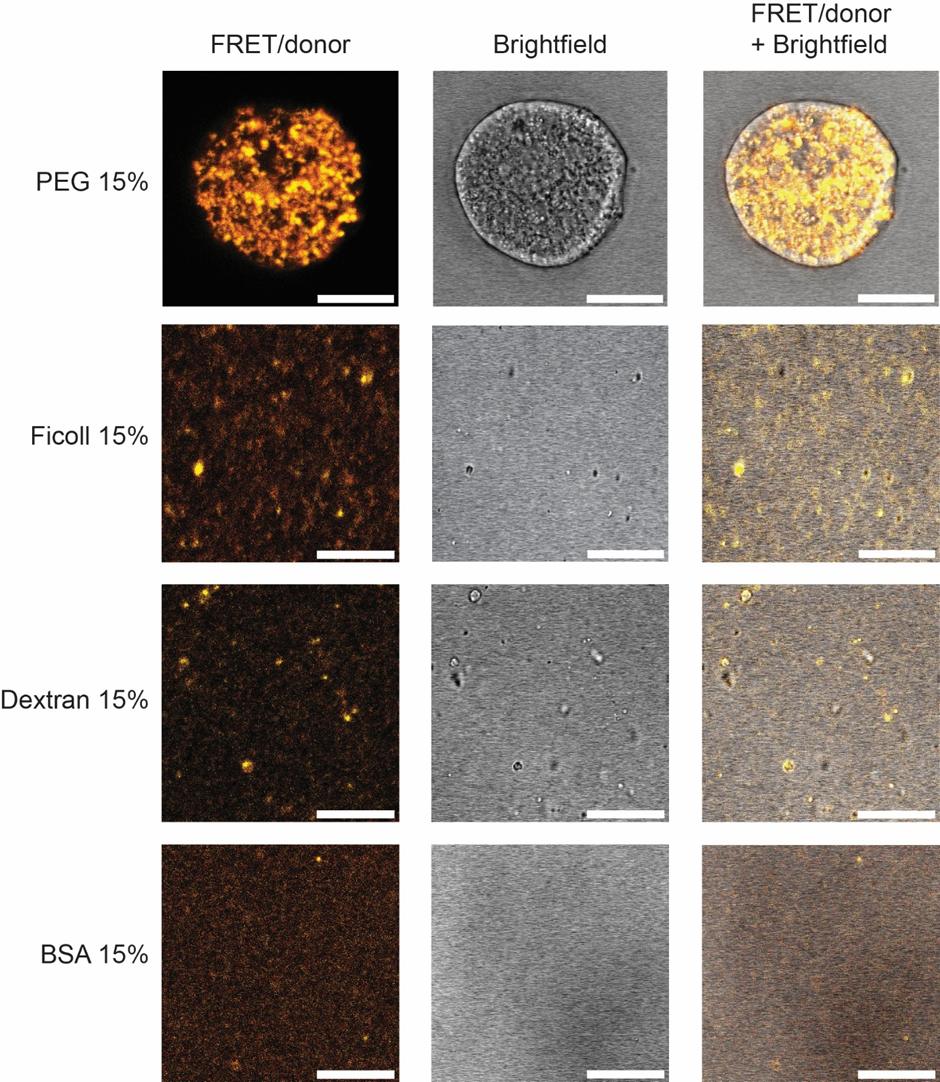


**Fig. S2.** **Effect of polymeric crowders on mHttex1-VC aggregation.** PEG 8000, Ficoll PM70, and Dextran 40 kDa induce different aggregate structures than in the presence of BSA. PEG and dextran induce droplets containing mHttex1-VC aggregates as visualized by superimposing the fluorescence channel on the brightfield channel (right). Scale bar 20 µm.


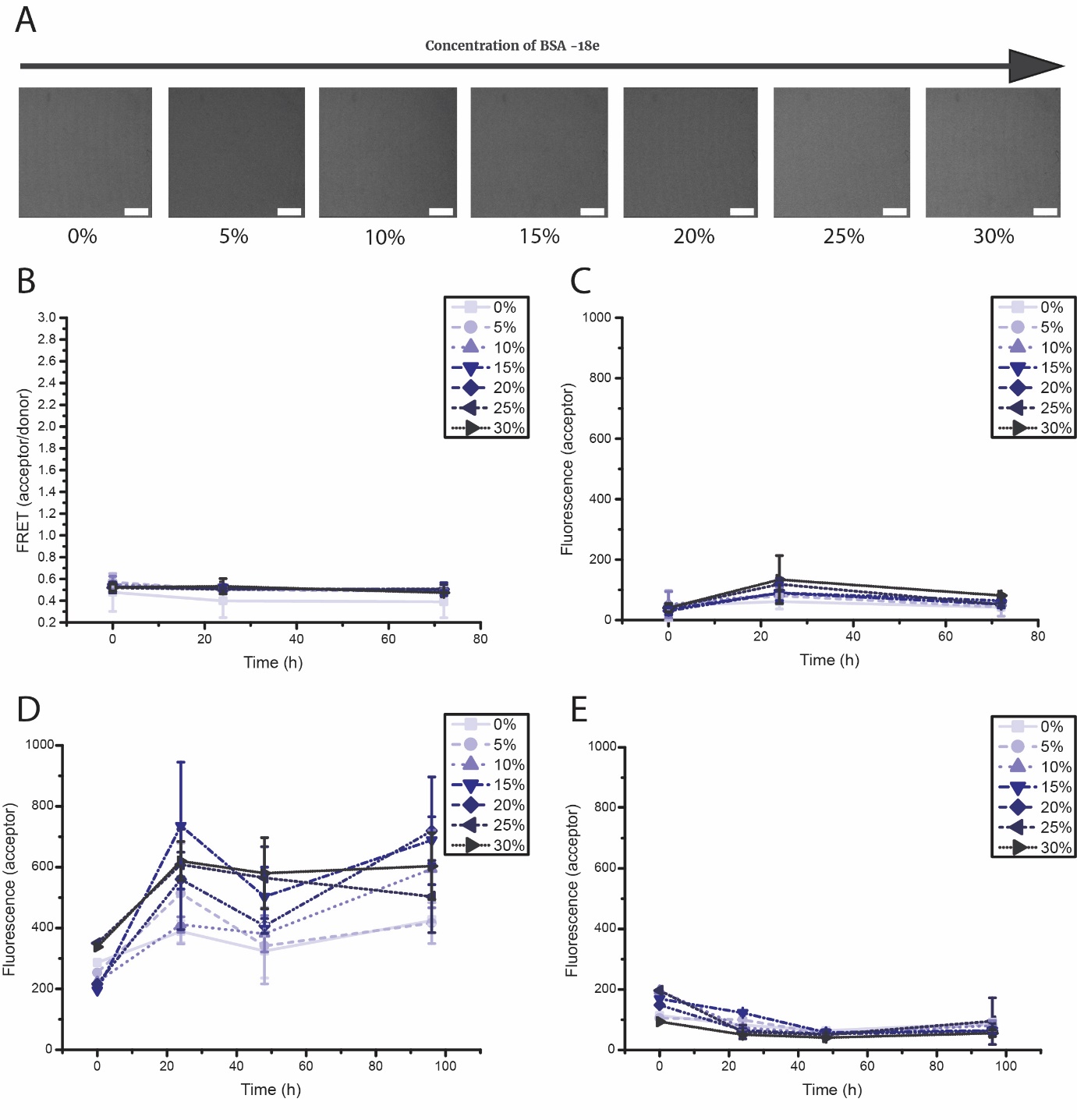


**Fig. S3.** **BSA crowding increases mHttex1-VC density in aggregates as assessed from direct acceptor excitation** and VC control. (A) Spinning disc confocal images of VC in different concentration of BSA, scale bar is 20 μm, (B) FRET/donor ratios and (C) acceptor fluorescence of VC in different BSA concentration versus time, (D) mHttex1-VC direct acceptor excitation and fluorescence in the aggregates, and (E) outside of visible aggregates. Error bars are s.d. over three independent replicates.


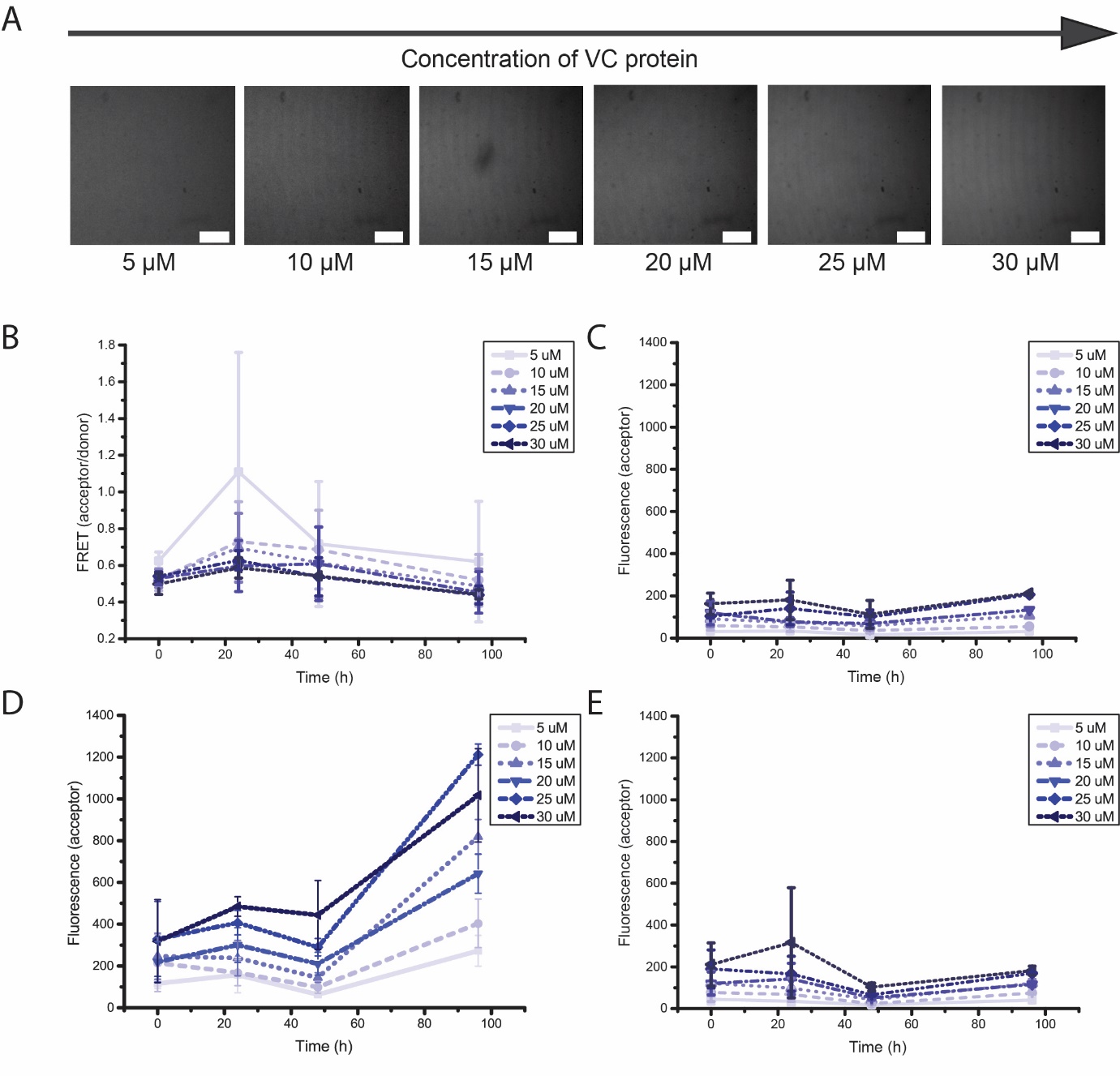


**Fig. S4.** **mHttex1-VC concentration increases aggregate density as assessed from direct acceptor excitation** and VC control. (A) Spinning disc confocal images of different VC concentrations showing absence of foci, scale bar is 20 μm, (B) FRET/donor ratios and (C) direct acceptor excitation of different VC concentrations in time, (D) mHttex1-VC direct acceptor excitation in the aggregates showing an increase in density with concentration, and (E) outside of visible aggregates showing a minor concentration dependence. Error bars are s.d. over three independent replicates.


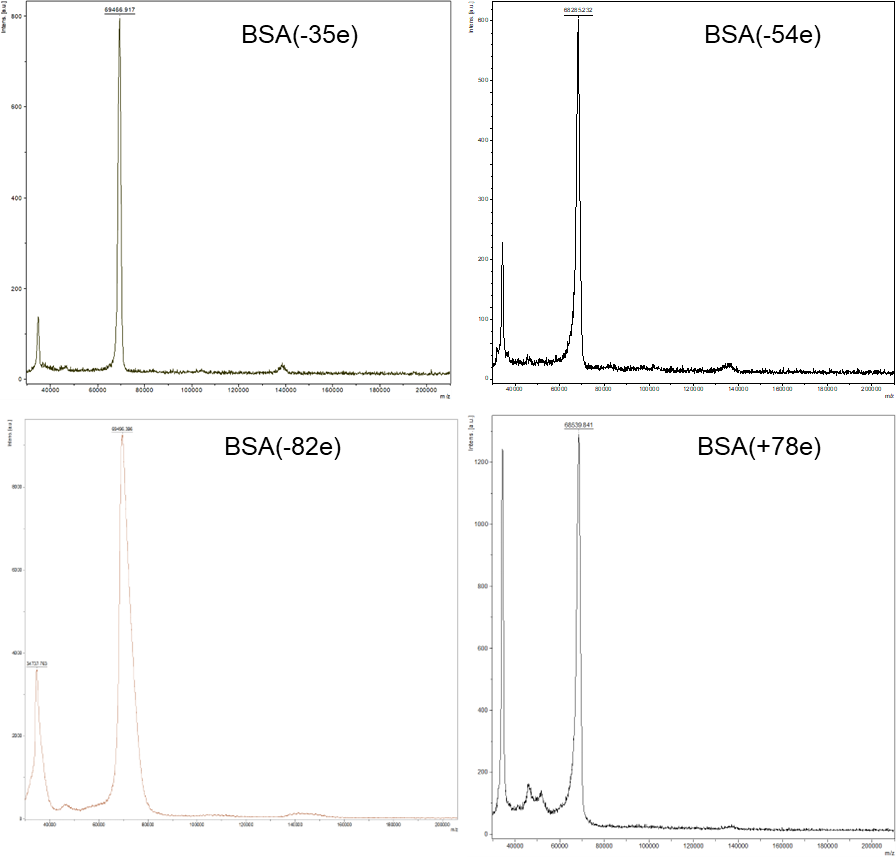


**Fig. S5.** **MALDI-TOF analysis of surface-modified BSAs.** Measured mass: -35e BSA: 69466 Da, -54e BSA: 68285 Da, -82e BSA: 69496 Da, +78e BSA: 68539 Da. In our experience, wild-type BSA displays batch-dependent 400 Da variation. This implies an error of ~±3e in the charge calculation. Theoretical mass: -35e BSA: 69.2 kDa, -54e BSA: 68.2 kDa, -82e BSA: 69.5 kDa, +78e BSA: 68.5 kDa. Some additional species can be seen, which correspond to multiply-charged oligomeric species.


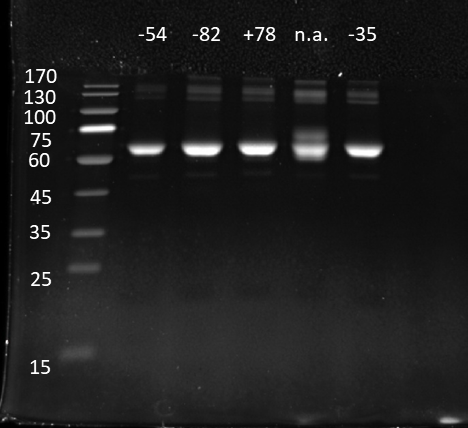


Figure S6. SDS PAGE of the modified BSAs showing single main band. Additional oligomeric species can be seen on top, as also observed in MALDI or DLS.


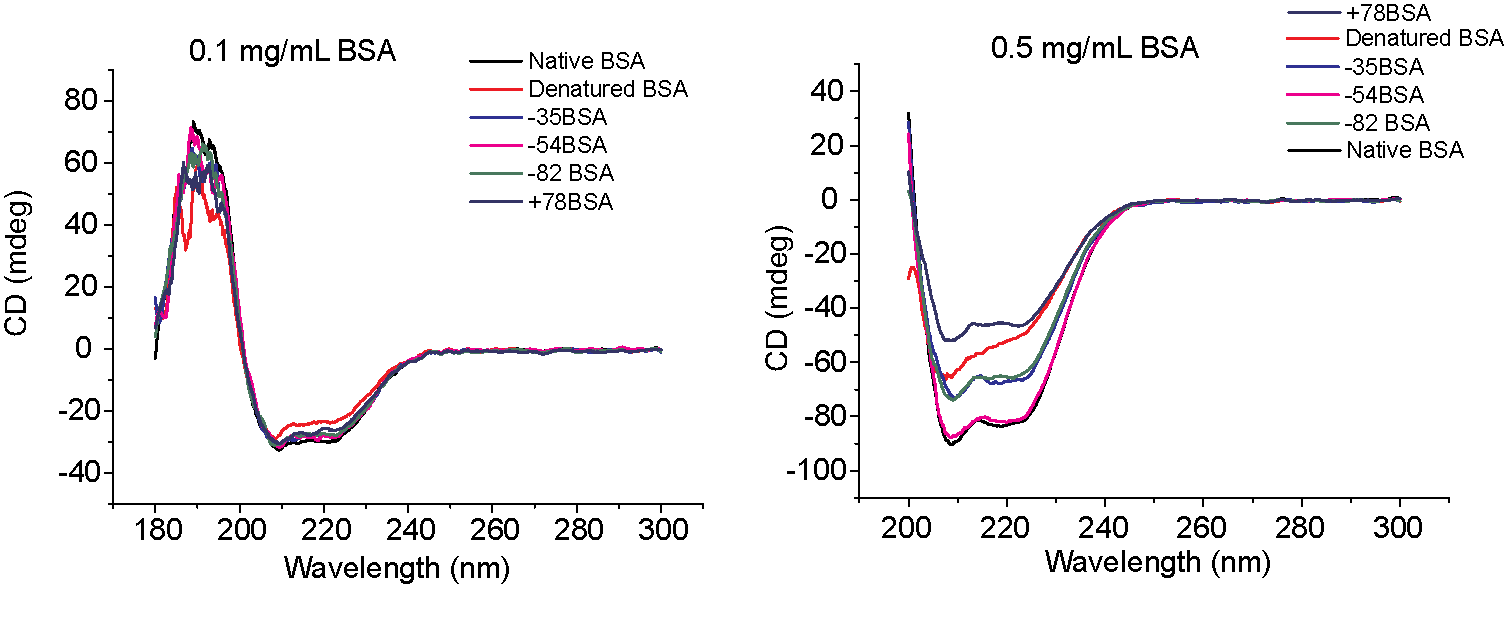


**Figure S7. Circular dichroism of the BSAs at 0.1 and 0.5 mg/mL BSA in NaPi buffer at 25 °C**. Intensities were variable for 0.5 mg/mL. This, in combination with the observation that the cuvettes were challenging to clean, suggests that the modified BSAs adsorb to the walls of the cuvettes. Denatured BSA shows a change in the shape of the CD spectrum, implying that its secondary structure is concentration dependent.


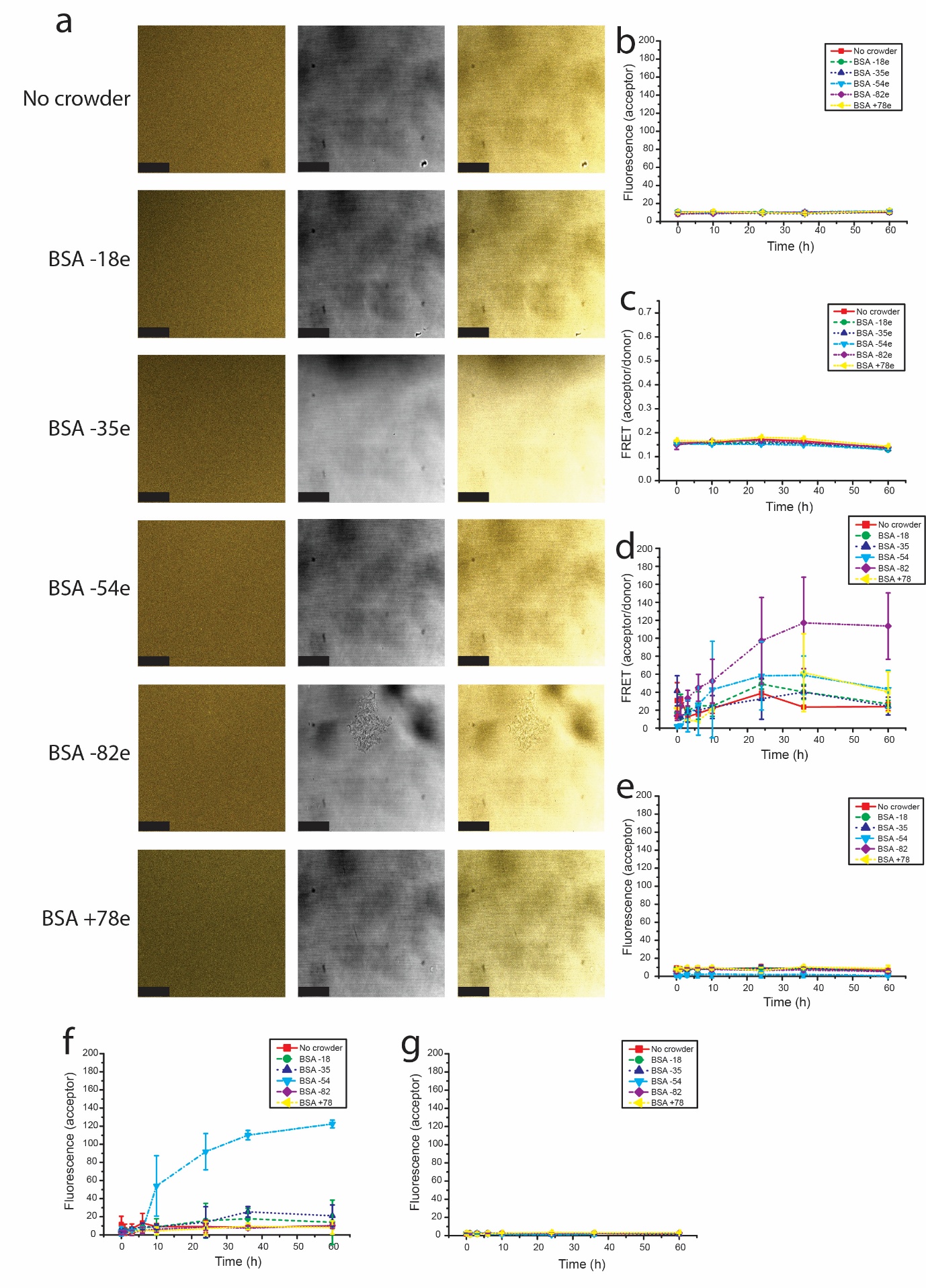


**Fig. S8.** **Control experiments showing that the VC domain by itself does not follow mHttex1-VC behaviour, and the comparison of mHttex1-VC direct acceptor excitation inside and outside visible aggregates with different surface-modified BSAs.** (A) Representative images of VC protein in the absence of crowder in PBS, pH 7.4, and with different BSAs at 15% w/w. Left column displays the FRET/Donor ratiometric image, middle column the brightfield channel, and on the right the superimposed FRET/donor and brightfield channels. The data show that the conditions do not lead to VC aggregation. Of note, BSA -82e aggregates can be clearly distinguished in the brightfield channel. (B) Direct acceptor excitation and fluorescence, showing the absence of any effect of the crowders on the VC domain. (C) FRET/donor ratios of VC in different BSAs, demonstrating lack of FRET increase, (D) mHttex1-VC direct acceptor excitation inside the aggregates in different BSAs at 15% w/w, PBS, pH 7.4, showing time-dependent increase in intensity, and (E) outside of visible aggregates. (F) mHttex1-VC direct acceptor excitation inside the aggregates, but in 10 mM NaPi, pH 7.4, and (G) outside of visible aggregates. Error bars are s.d. over three independent replicates.


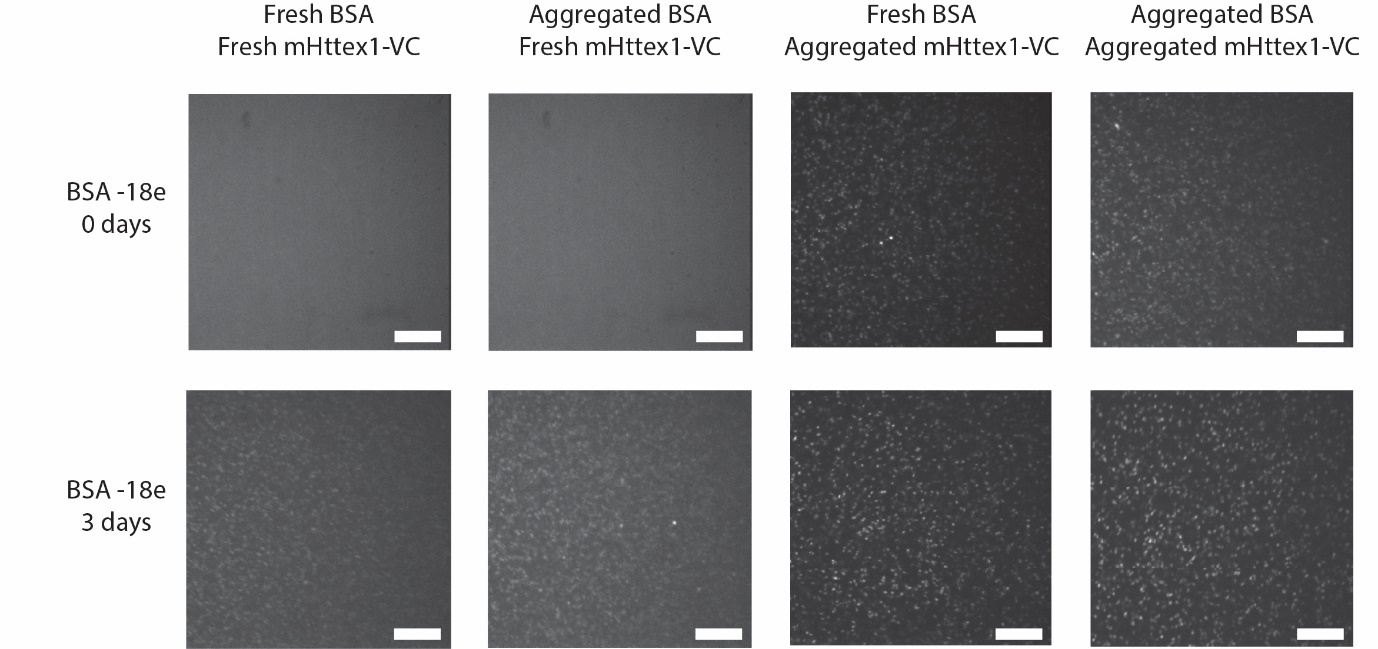


**Fig. S9. Test for coaggregation or seeding by BSA -18e.** The experiment was performed as in Figure 5a, using BSA -18e instead of BSA -82e, showing the absence of enhanced aggregation when using the aged BSA sample. Scale bar is 20 μm.


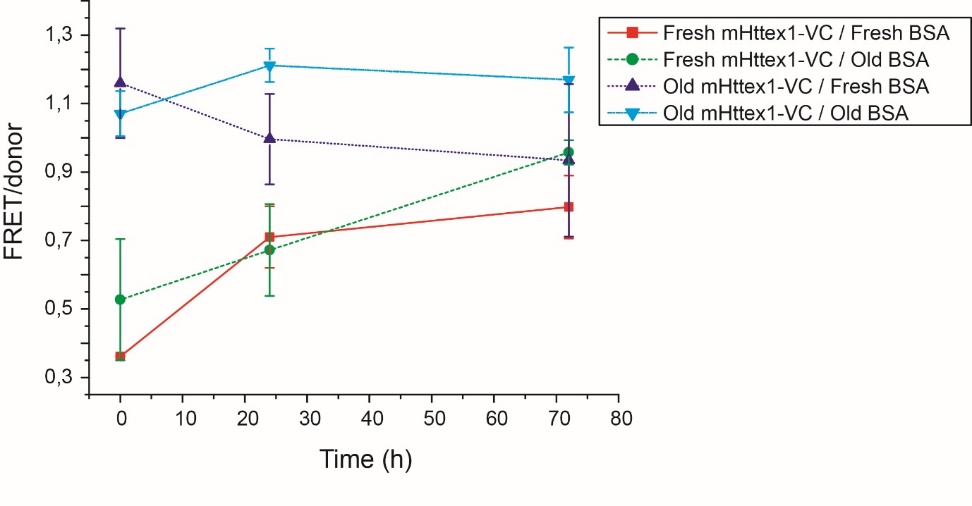


**Fig. S10. Analysis of FRET/donor in mHttex-VC aggregates corresponding to Figure 5A.** The FRET/Donor for all conditions becomes more similar over time as the final solutions are similar, despite the aggregate size differences. As expected, old mHttex1-VC starts with a higher FRET than fresh mHttex1-VC. Final conditions: 10 µM mHttex1-VC, 5% BSA -82e, PBS, pH 7.4 (data are three independent replicates, ±s.d.).


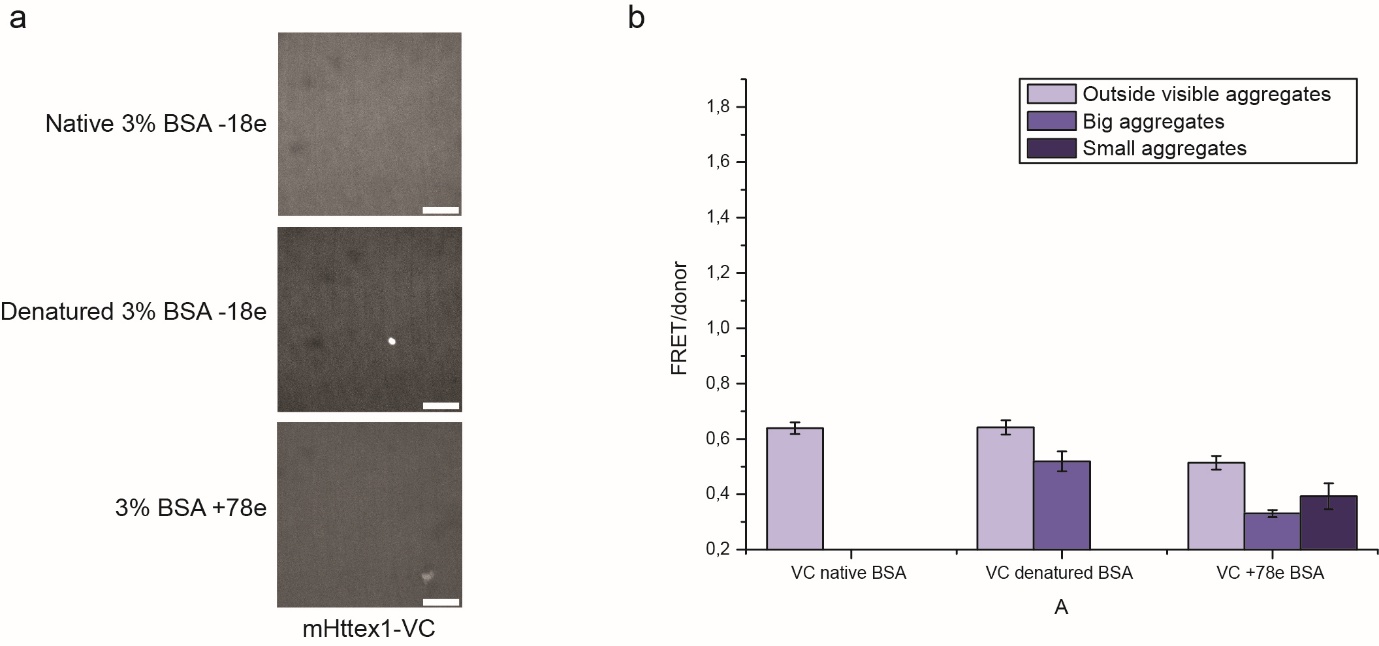


**Fig. S11. VC control with 3% w/w native and denatured BSA -18e and BSA +78e.** (a) Spinning disc confocal microscopy images of VC with 3% w/w BSA -18e, heat-denatured BSA -18e, and BSA +78e, (b) bar graphs of FRET/donor ratios in the sporadic visible foci and outside visible foci. Error bars are s.d. over three independent replicates.


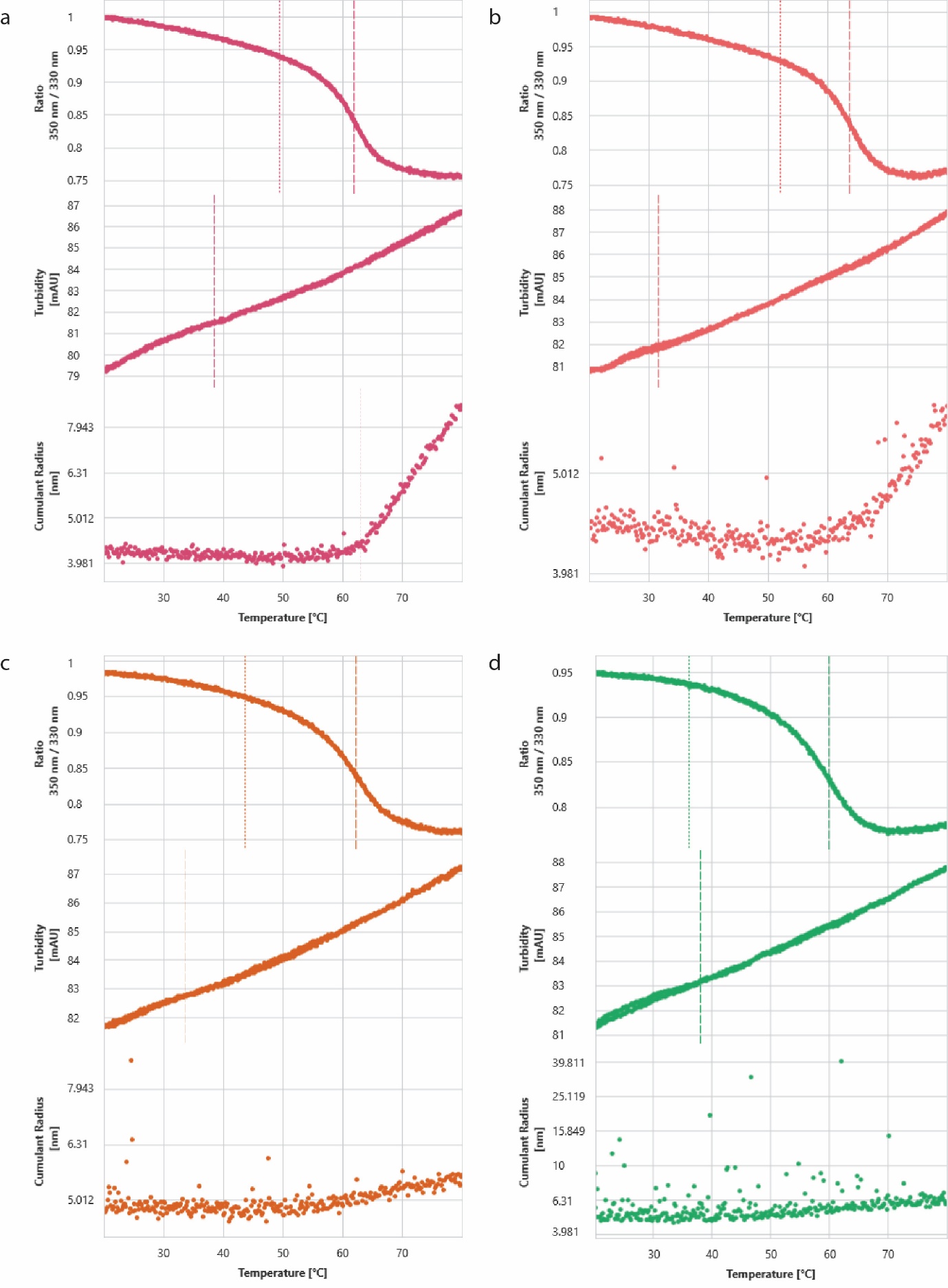


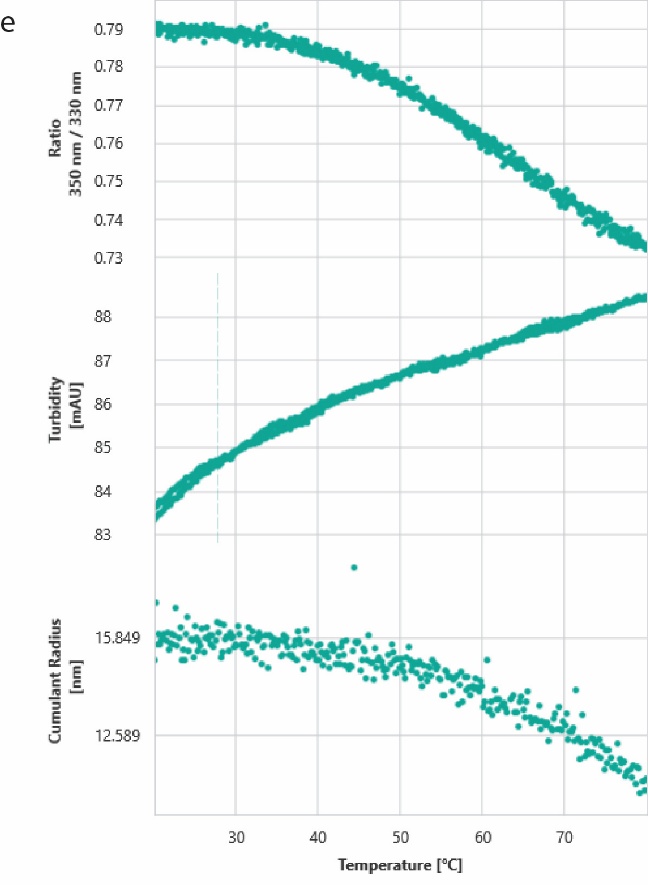


**Fig. S12. Thermal denaturation of different surface-charged BSAs in PBS pH 7.4 – data from Prometheus Panta, representing protein stability, turbidity and cumulant radius (nm).** 2 mg/mL (a) BSA -18e, (b) BSA -35e, (c) BSA -54e, (d) BSA -82e, (e) BSA +78e.


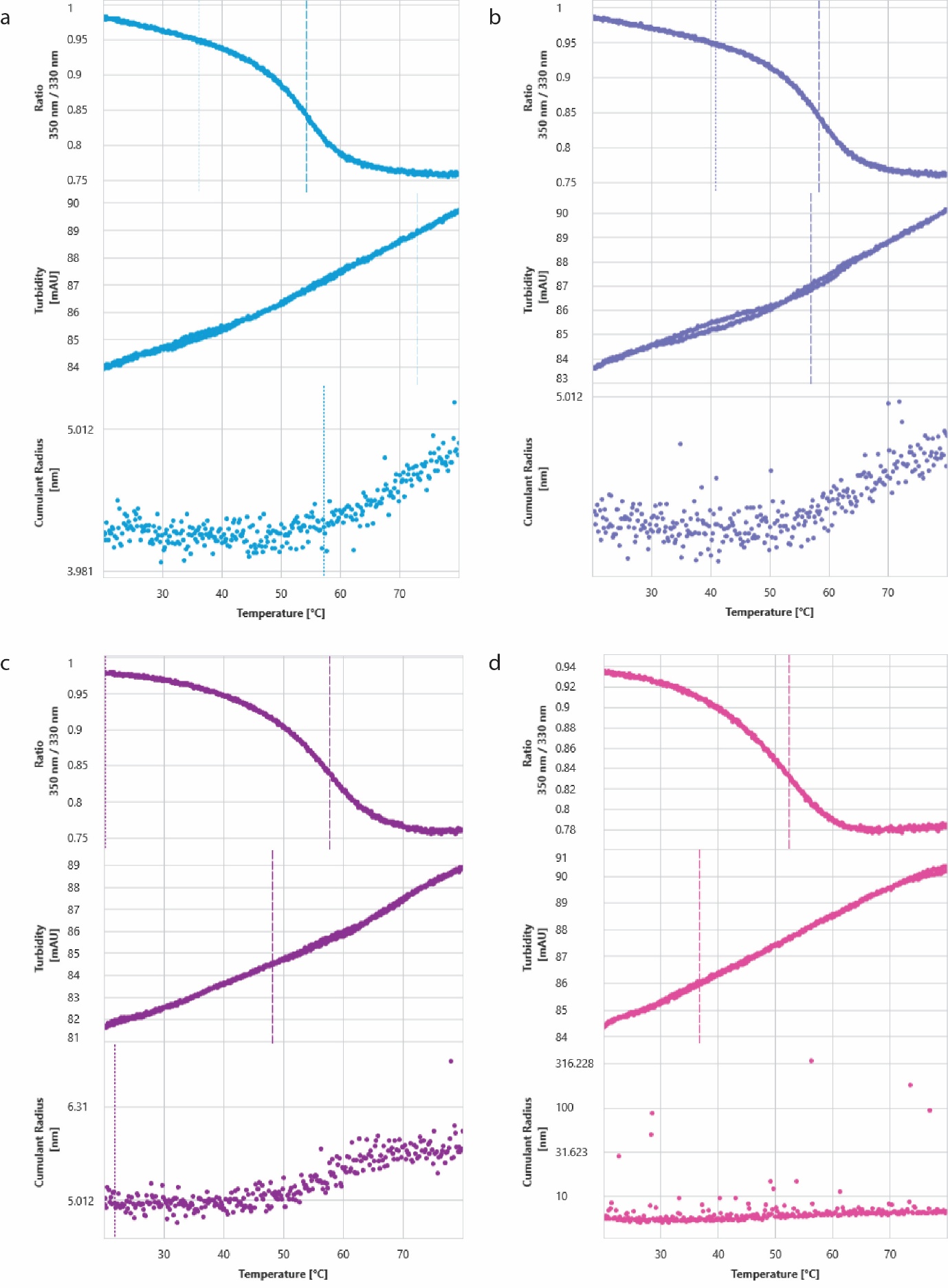


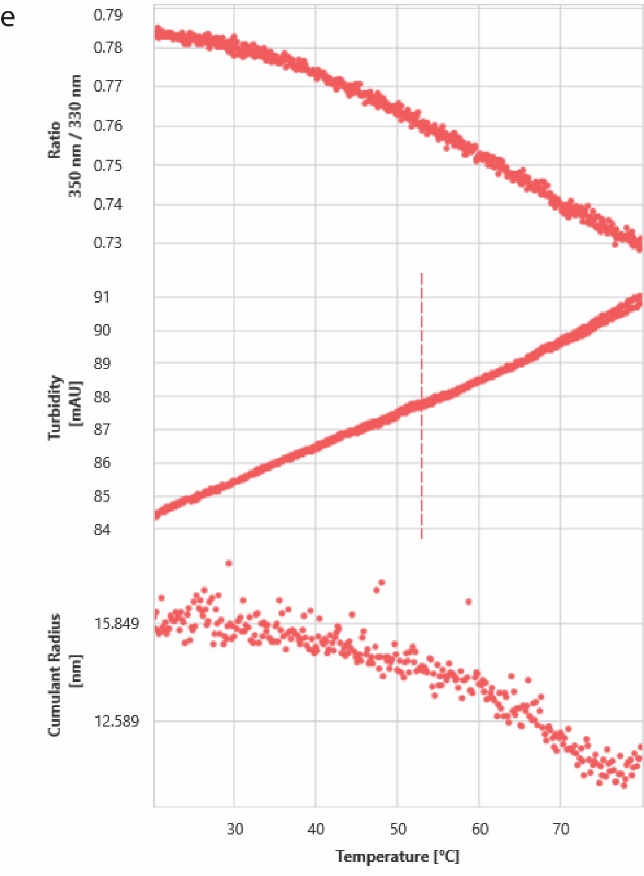


**Fig. S13. Thermal denaturation of different surface-charged BSAs in 10 mM NaPi pH 7.4 – data from Prometheus Panta, representing protein stability, turbidity and cumulant radius (nm).** 2 mg/mL (a) BSA -18e, (b) BSA -35e, (c) BSA -54e, (d) BSA -82e, (e) BSA +78e.


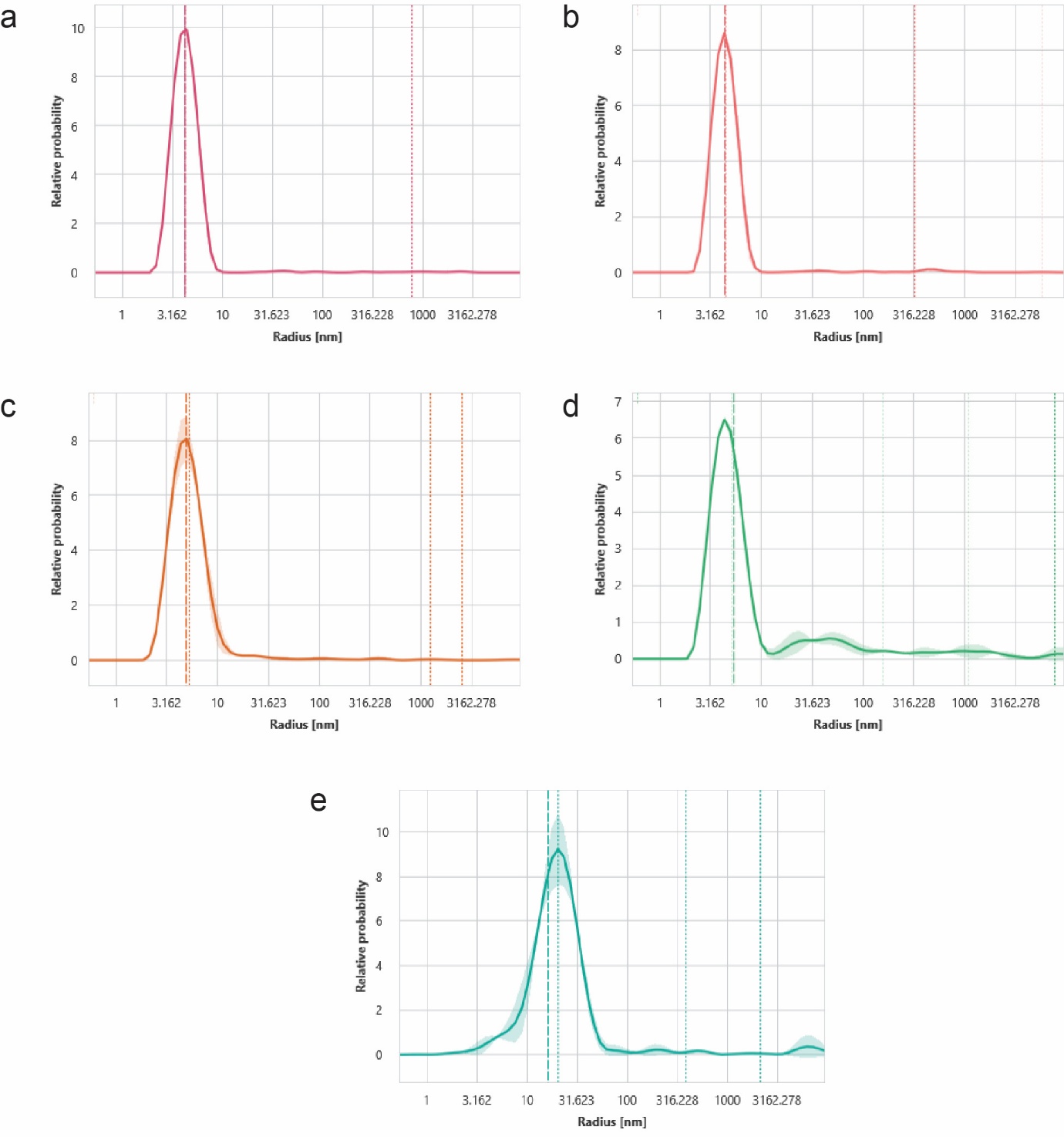


**Fig. S14. Prometheus Panta DLS analysis of different modified BSAs in PBS,
pH 7.4.** 2 mg/mL (a) BSA -18e, (b) BSA -35e, (c) BSA -54e, (d) BSA -82e, (e) BSA +78e.


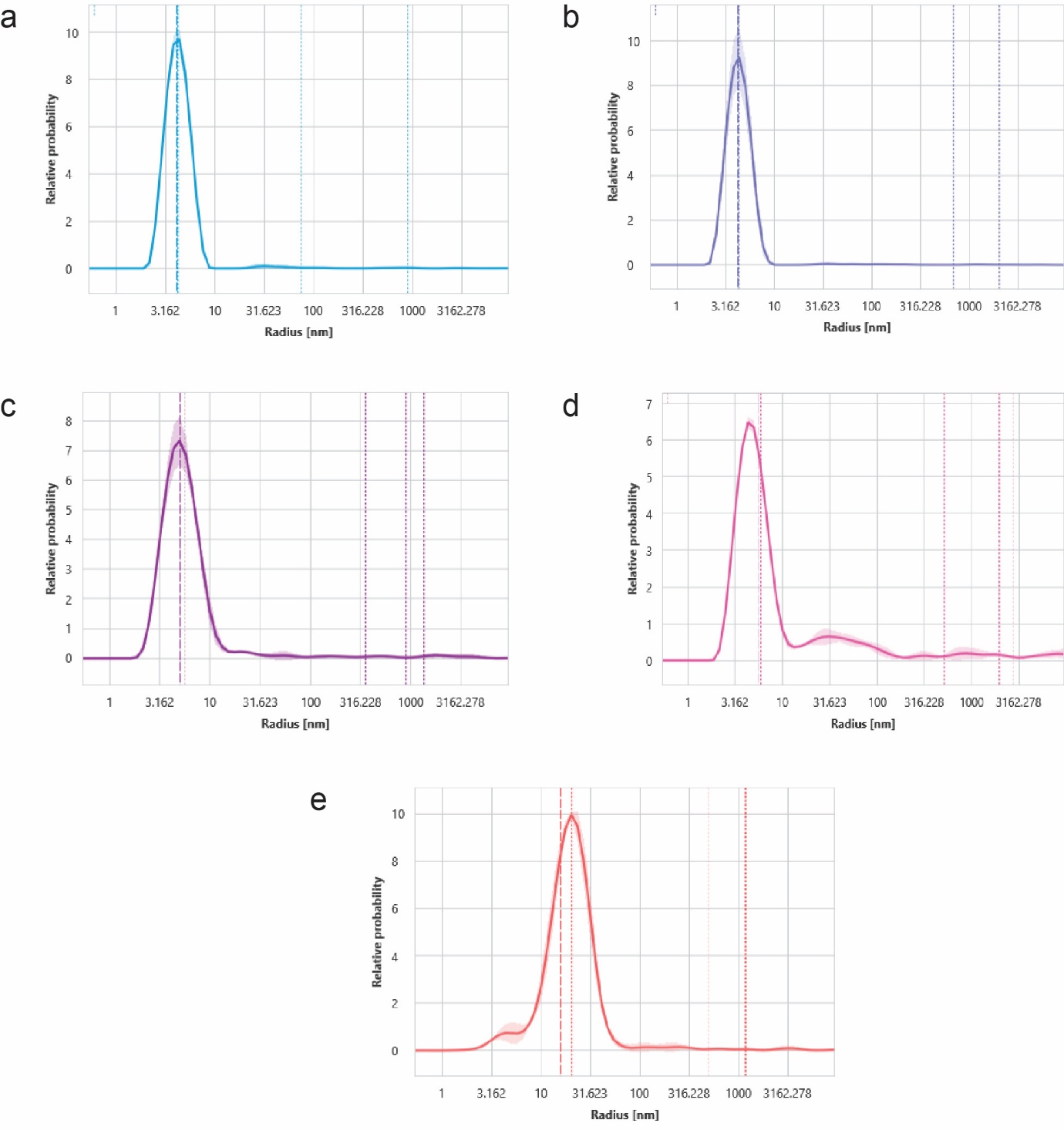


**Fig. S15. Prometheus Panta DLS analysis of different modified BSAs in
10 mM NaPi, pH 7.4.** 2 mg/mL (a) BSA -18e, (b) BSA -35e, (c) BSA -54e, (d) BSA -82e, (e) BSA +78e.

**DNA sequences.**

**MBP-mHttex1-mVenus/mCherry**

ATGCATCATCATCACCATCATAAAATCGAAGAAGGTAAACTGGTAATCTGGATTAACGGCGATAAAGGCTATAACGGTCTCGCTGAAGTCGGTAAGAAATTCGAGAAAGATACCGGAATTAAAGTCACCGTTGAGCATCCGGATAAACTGGAAGAGAAATTCCCACAGGTTGCGGCAACTGGCGATGGCCCTGACATTATCTTCTGGGCACACGACCGCTTTGGTGGCTACGCTCAATCTGGCCTGTTGGCTGAAATCACCCCGGACAAAGCGTTCCAGGACAAGCTGTATCCGTTTACCTGGGATGCCGTACGTTACAACGGCAAGCTGATTGCTTACCCGATCGCTGTTGAAGCGTTATCGCTGATTTATAACAAAGATCTGCTGCCGAACCCGCCAAAAACCTGGGAAGAGATCCCGGCGCTGGATAAAGAACTGAAAGCGAAAGGTAAGAGCGCGCTGATGTTCAACCTGCAAGAACCGTACTTCACCTGGCCGCTGATTGCTGCTGACGGGGGTTATGCGTTCAAGTATGAAAACGGCAAGTACGACATTAAAGACGTGGGCGTGGATAACGCTGGCGCGAAAGCGGGTCTGACCTTCCTGGTTGACCTGATTAAAAACAAACACATGAATGCAGACACCGATTACTCCATCGCAGAAGCTGCCTTTAATAAAGGCGAAACAGCGATGACCATCAACGGCCCGTGGGCATGGTCCAACATCGACACCAGCAAAGTGAATTATGGTGTAACGGTACTGCCGACCTTCAAGGGTCAACCATCCAAACCGTTCGTTGGCGTGCTGAGCGCAGGTATTAACGCCGCCAGTCCGAACAAAGAGCTGGCAAAAGAGTTCCTCGAAAACTATCTGCTGACTGATGAAGGTCTGGAAGCGGTTAATAAAGACAAACCGCTGGGTGCCGTAGCGCTGAAGTCTTACGAGGAAGAGTTGGTGAAAGATCCGCGTATTGCCGCCACTATGGAAAACGCCCAGAAAGGTGAAATCATGCCGAACATCCCGCAGATGTCCGCTTTCTGGTATGCCGTGCGTACTGCGGTGATCAACGCCGCCAGCGGTCGTCAGACTGTCGATGAAGCCCTGAAAGACGCGCAGGGTGGTGGTGGTGGTGGTGAAAACCTGTACTTCCAAGGCGCTACCTTGGAGAAATTGATGAAGGCTTTCGAATCCTTGAAGTCCTTTCAACAACAGCAGCAACAACAGCAACAGCAACAACAACAACAGCAGCAACAACAACAGCAACAGCAACAACAGCAGCAACAACAACAGCAGCAACAGCAACAACAACAGCAACAGCAACAACAGCAACAGCAGCAACCACCACCACCACCACCTCCACCTCCACCACCTCAATTGCCACAACCACCACCTCAAGCTCAACCATTATTACCACAACCTCAGCCACCACCTCCTCCACCACCACCACCACCTGGTCCAGCTGTTGCAGAAGAACCATTGCATAGACCAGGTTCTTTGGTTTCTAAAGGTGAAGAGTTGTTCACTGGTGTTGTTCCAATTTTGGTTGAATTGGATGGTGATGTTAACGGCCATAAGTTTTCTGTTTCTGGTGAAGGTGAGGGTGATGCTACTTATGGTAAATTGACTTTGAAGTTGATCTGCACCACAGGTAAATTGCCAGTTCCATGGCCAACTTTGGTTACTACTTTAGGTTACGGCTTGCAATGTTTTGCTAGATACCCAGATCATATGAAGCAACACGATTTCTTCAAATCCGCTATGCCAGAAGGTTACGTTCAAGAAAGAACTATCTTCTTCAAGGACGACGGTAACTACAAAACTAGAGCTGAAGTTAAGTTCGAAGGTGATACCTTGGTTAACAGGATTGAATTGAAGGGCATCGATTTCAAAGAGGATGGTAACATTTTGGGTCACAAGTTGGAGTACAACTACAACTCTCATAACGTTTACATTACCGCCGACAAGCAAAAGAATGGTATTAAGGCTAACTTCAAGATCAGGCACAACATTGAAGATGGTGGTGTTCAATTGGCTGATCACTATCAACAAAACACCCCAATTGGTGATGGTCCAGTTTTGTTGCCAGATAACCATTACTTGTCCTACCAGTCCAAGTTGTCTAAAGACCCAAACGAAAAAAGGGATCACATGGTTTTGTTGGAATTTGTTACAGCTGCTTCCGGTGATAACATGGCCATTATCAAAGAATTTATGAGGTTCAAGGTCCACATGGAAGGTTCTGTTAATGGTCACGAATTTGAGATTGAAGGTGAAGGCGAAGGTAGACCATATGAAGGTACTCAAACTGCTAAACTGAAGGTTACAAAAGGTGGTCCATTGCCATTTGCTTGGGATATTTTGTCTCCACAATTCATGTACGGTTCTAAGGCTTATGTAAAACACCCAGCTGATATCCCAGATTACTTGAAGTTGTCATTTCCAGAGGGTTTCAAGTGGGAAAGAGTTATGAATTTCGAGGATGGTGGCGTTGTTACTGTTACTCAAGATTCTTCATTACAGGACGGTGAGTTTATCTACAAGGTTAAGTTGAGAGGTACGAACTTTCCATCTGATGGTCCTGTTATGCAAAAAAAGACTATGGGTTGGGAAGCCTCTTCTGAAAGAATGTATCCAGAAGATGGCGCTTTGAAAGGTGAAATCAAACAAAGGTTGAAATTGAAGGACGGTGGTCATTATGATGCCGAAGTTAAGACTACTTACAAGGCTAAAAAGCCAGTTCAATTACCAGGTGCTTACAACGTCAACATCAAGTTGGATATCACTTCCCACAACGAAGATTACACCATCGTTGAACAATACGAAAGAGCTGAGGGTAGACATTCTACTGGTGGTATGTAA

**mVenus/mCherry**

ATGGTTAGTAAAGGTGAAGAATTATTTACGGGGGTAGTGCCGATTCTTGTGGAACTTGATGGCGATGTTAACGGACACAAGTTTTCGGTCAGCGGGGAGGGGGAAGGGGATGCTACTTATGGGAAACTGACGCTGAAATTAATCTGTACAACGGGTAAGCTGCCCGTGCCGTGGCCCACGTTAGTAACCACGTTAGGGTATGGCTTGCAGTGTTTTGCCCGTTATCCCGACCACATGAAACAACATGACTTTTTTAAGTCGGCCATGCCTGAGGGTTATGTGCAAGAACGCACAATCTTTTTCAAAGATGATGGCAATTACAAAACACGCGCCGAAGTCAAGTTCGAAGGCGACACCTTGGTCAACCGCATTGAGTTAAAAGGTATCGATTTCAAAGAAGACGGTAACATTTTAGGCCATAAACTTGAATACAACTACAATTCACATAATGTATATATCACGGCAGACAAACAAAAGAATGGAATTAAGGCTAACTTTAAGATTCGCCATAACATCGAGGACGGTGGAGTACAGTTAGCAGACCACTACCAACAGAACACGCCAATCGGAGATGGCCCAGTTCTTTTGCCTGATAACCACTACTTGTCATACCAAAGCAAGCTGTCCAAAGACCCAAATGAAAAACGTGATCACATGGTGCTTCTGGAATTTGTGACCGCCGCCTCTGGGGATAACATGGCCATCATCAAAGAGTTTATGCGCTTTAAGGTCCACATGGAGGGTAGCGTGAACGGACACGAATTCGAGATTGAAGGGGAGGGGGAAGGACGCCCCTACGAGGGCACGCAAACGGCAAAATTAAAGGTAACTAAGGGTGGTCCTCTTCCTTTCGCATGGGATATCTTAAGCCCTCAGTTTATGTATGGGAGTAAGGCTTATGTAAAGCACCCTGCAGACATTCCAGATTATCTGAAGTTGAGTTTTCCCGAAGGGTTTAAATGGGAACGTGTTATGAACTTTGAGGACGGGGGTGTAGTCACGGTCACTCAAGACAGCAGCTTACAAGATGGTGAGTTTATCTATAAAGTCAAGTTGCGCGGAACCAACTTTCCATCGGATGGCCCTGTCATGCAAAAAAAAACGATGGGTTGGGAAGCATCGTCCGAACGCATGTACCCCGAGGACGGTGCCCTGAAGGGGGAAATTAAACAACGCTTGAAACTTAAAGATGGAGGTCACTATGATGCAGAGGTCAAGACAACATACAAGGCCAAGAAACCAGTACAGCTGCCGGGTGCCTATAATGTGAATATCAAGTTGGACATTACATCCCACAATGAGGACTATACGATTGTAGAGCAATACGAGCGCGCTGAGGGTCGTCACTCTACCGGCGGTATGGAGAATTTGTACTTCCAGGGACATCATCATCACCATCATTAA
